# Updates on the Translational Value of Idiopathic Pulmonary Fibrosis Models Across Species: Deeper Insights Through Comparative Pathology

**DOI:** 10.3390/biom16101519

**Published:** 2026-10-19

**Authors:** Sara Gargiulo, Matteo Gramanzini, Denise Bonente, Virginia Barone

**Affiliations:** 1 Institute of Clinical Physiology, National Research Council, Via Fiorentina 1, 53100 Siena, Italy; 2 Institute of Crystallography, National Research Council, Strada Provinciale 35d, n. 9, Montelibretti, 00010 Rome, Italy; matteo.gramanzini@cnr.it; 3 Department of Life Sciences, University of Siena, 53100 Siena, Italy; denise.bonente2@unisi.it; 4 Department of Molecular and Developmental Medicine, University of Siena, 53100 Siena, Italy; virginia.barone@unisi.it

**Keywords:** idiopathic pulmonary fibrosis (IPF), comparative medicine, spontaneous models, experimental models, genetically modified models, imaging, histopathology

## Abstract

Over the past few decades, advances in comparative pathology have strengthened the potential translational value of animal models of Idiopathic Pulmonary Fibrosis (IPF). The integration of human-relevant diagnostic biomarkers into preclinical studies, such as advanced imaging techniques and standardized histopathological methods, has improved the comparability of experimental findings with human disease. Naturally occurring fibrotic lung diseases in some breeds of companion dogs and cats and induced disease models in laboratory rodents show varying degrees of similarity to human IPF. They represent valuable comparative medicine tools to better understand disease mechanisms and test new therapies, each with advantages and limitations. Critically evaluating their complementarity is essential for gaining reliable insights into disease processes through preclinical animal studies and for developing effective treatments. This comprehensive review aims to update current knowledge of these animal models of IPF, focusing on comparative pathology, imaging, and histological features and evaluating their respective strengths, limitations, and translational relevance within the conceptual framework of “One Health, One Medicine, One Pathology”. By exploiting convergent pathophysiological processes across species, this interdisciplinary collaborative paradigm aims to promote the effective use of model organisms in biomedical research and foster mutual benefits for both humans and animals.

## 1. Introduction

Idiopathic pulmonary fibrosis (IPF) is the most common form of chronic, progressive, and irreversible interstitial lung disease (ILD), which primarily involves the pulmonary interstitium, the tissue space between alveolar epithelial cells, capillary endothelial cells, and the intervening basement membrane. It affects approximately 3 million people worldwide, with incidence varying across Asia, North America, and Europe. IPF is more prevalent among men and postmenopausal women, and its incidence increases with age [1,2,3].

The precise aetiology of IPF remains uncertain. The prevailing hypothesis suggests that repeated mild lung injuries of various origins trigger persistent interactions between inflammatory and fibrotic processes. Several environmental exposures and potential risk factors have been implicated in disease development, including cigarette smoking, air pollution, occupational exposures, bacterial and viral infections, and comorbidities such as gastroesophageal reflux disease, emphysema, cardiovascular diseases, and autoimmune disorders. A family history of IPF may further suggest a genetic predisposition involving genes that regulate mucociliary clearance and host defence, such as MUC5B (particularly the rs35705950 variant), TOLLIP, and OBFC1, as well as rare genetic alterations affecting telomere maintenance and surfactant secretion [3,4,5].

IPF is associated with worsening quality of life and a median survival of approximately 3–5 years after diagnosis [3,4,5]. Over the past decade, antifibrotic treatments, including pirfenidone, nintedanib, and nerandomilast, have improved disease management by slowing its progression; however, the prognosis remains poor [6,7].

Consequently, several potential new therapeutic options are under investigation [8].

In this context, clinical guidelines emphasize the importance of early diagnosis through a multidisciplinary, multiparametric assessment integrating clinical, histopathological, and imaging data, particularly to support the differential diagnosis of other ILDs and underlying conditions [9]. These approaches are also valuable for monitoring disease progression and treatment response. By contrast, molecular tools such as genomic classifiers, serum tests, and proteomic biomarkers are not yet recommended for routine clinical use and require further validation to establish their sensitivity, specificity, and incremental clinical utility [3,10].

The histopathological hallmark of IPF is the usual interstitial pneumonia (UIP) pattern, characterized by bronchiectasis and bronchiolectasis, emphysema, pleural and septal fibrosis. However, this pattern of lung injury is not specific to IPF and may also be observed in other ILDs of known aetiology, including those associated with infections, toxic, exposures or autoimmune diseases. Therefore, differential diagnosis requires the integration of clinical, imaging and histopathological findings [11].

Advanced techniques, such as high-resolution computed tomography (HRCT), ultrashort echo time (UTE) or hyperpolarized ^129^Xe magnetic resonance imaging (MRI), positron emission tomography (PET), histological fractal morphometry, and transbronchial cryobiopsy, provide additional diagnostic and prognostic information to improve IPF assessment. Non-invasive imaging techniques may facilitate diagnosis and enable longitudinal monitoring of disease progression and treatment response, potentially reducing the need for surgical lung biopsy. When tissue sampling is required, transbronchial cryobiopsy represents a less invasive alternative to surgical lung biopsy [3,9,12,13,14,15,16,17].

HRCT is currently the first-line imaging modality for distinguishing IPF from other common fibrosing interstitial lung diseases. The HRCT features of the UIP pattern include traction bronchiectasis and bronchiolectasis, which appear radiologically as irregularly dilated and distorted airways resulting from surrounding fibrotic retraction; reticulation (a network of fine linear opacities reflecting thickening of the interstitial structures); ground-glass attenuation (a subtle increase in lung opacity through which the pulmonary vessels and underlying bronchial structures remain visible); and honeycombing (clustered cystic dilation of peripheral airspaces surrounded by fibrosis) [1,3,9].

These imaging features correlate well with the histologic findings of IPF, and recent clinical recommendations indicate that invasive histological confirmation may be unnecessary in selected cases when a defined UIP pattern is identified on HRCT [3,9,12].

However, as with histopathological findings, HRCT features may overlap among different lung diseases, highlighting the limitations of imaging alone and the importance of a multidisciplinary approach to diagnosis.

Animal models remain essential for elucidating IPF pathogenesis and identifying disease-specific features that may distinguish it from other ILDs, although no single model fully recapitulates the human disease.

Despite the availability of a broad range of experimental animal models, rodents, particularly aged mice, remain the most widely used models for preclinical mechanistic studies and drug testing. This is largely due to their amenability to genetic manipulation, high reproducibility, cost-effectiveness, and shorter experimental timescales compared with larger mammalian models [18]. Recent advances in small-animal imaging enable the noninvasive monitoring of disease progression and treatment response using imaging modalities that are comparable across species. However, these preclinical models also present several limitations. Clinically relevant outcome measures, such as lung function assessment, are not readily applicable; therapeutic interventions are often tested prophylactically, before the development of established and persistent lung fibrosis; and experimental protocols may vary considerably across laboratories.

Beyond traditional experimentally induced models of pulmonary fibrosis in laboratory mice and rats, spontaneous fibrotic lung diseases in certain breeds of dogs and cats may represent valuable comparative models, offering complementary insights that could help address some of the limitations of conventional rodent models.

The recognition that evolutionarily conserved pathogenetic mechanisms across species can provide synergistic insights into both human and veterinary medicine, with mutual benefits, has underpinned the concept of ‘One Medicine’ for more than a century [19].

This holistic “reverse translational” approach represents an important component of the broader “One Health” paradigm, which emphasizes the interconnectedness of the health of all living beings sharing the same environment, and may support the more rational and ethically informed use of animal models in biomedical research [20].

Crucially, recent advances in veterinary clinical practice and diagnostic techniques, such as imaging and image-guided biopsies, have made the implementation of multimodality approaches increasingly feasible in animal patients.

In particular, naturally occurring pulmonary fibrotic diseases in companion dogs and cats may recapitulate, to varying degrees, overlapping genetic susceptibilities, pathophysiological features, and environmental risk factors shared with human IPF, thereby providing insights into disease mechanisms and progression in the context of naturally occurring disease [21].

These models may also facilitate the evaluation of potential therapeutic strategies, including dosing regimens in animals with established fibrosis, and enable the study of progressive, irreversible idiopathic disease over a shorter lifespan than that of humans [21].

However, substantial interindividual variability in disease progression and clinical manifestations may limit the reproducibility of spontaneous models. Furthermore, differences in imaging practices and histopathological classification criteria between veterinary and human medicine may hinder the standardization and cross-species comparison of findings, posing a significant challenge to their translational application in research and therapeutic development.

Overall, the use of experimental or operative procedures that reflect clinical practice, such as translational imaging techniques and standardized histological assessments, in complementary animal models may enhance the comparability of preclinical findings with human disease while promoting methodological refinement in accordance with the 3Rs.

Notably, imaging enables noninvasive longitudinal assessment of disease onset, progression, and treatment response in experimental rodent models and spontaneously occurring fibrotic lung disease in companion dogs and cats, facilitating comparison with clinically relevant imaging features of human IPF. In murine models, serial imaging of the same animals reduces inter-animal variability and may decrease the number of animals required to detect biologically meaningful changes, thereby improving statistical power.

In companion dogs and cats, imaging also enables the characterization and repeated monitoring of naturally occurring disease in larger animals whose lung size and anatomy are closer to the human condition than in rodents, supporting closer alignment between veterinary and human diagnostic and prognostic approaches.

Moving beyond model selection based primarily on availability and existing expertise requires integrating induced and naturally occurring models and exploring alternative experimental approaches.

Since neither type of model fully recapitulates the complexity of human IPF, their complementary strengths should be leveraged to investigate distinct aspects of its pathobiology.

Comparative pathology requires multidisciplinary expertise to distinguish pathological similarities from differences between animal models and human IPF, particularly with regard to imaging and histological features [19].

Careful interpretation of interspecies findings, together with the adoption of harmonized diagnostic protocols, appropriate translational methodologies, and models that better reflect the characteristics of elderly patients, may improve the predictive value of animal studies and strengthen the evidence base before clinical trials.

Furthermore, investigating genetic and non-genetic risk factors underlying interspecies variation may also provide further insights into disease heterogeneity and inform the development of more personalized approaches to IPF.

These efforts may ultimately support the development of models with greater predictive value for clinical outcomes, in line with the “One Health, One Medicine, One Pathology” vision.

The aim of this review is therefore to critically evaluate the imaging and histopathological features of selected complementary animal models of IPF-like fibrosing lung disease that are translationally relevant and particularly well suited to experimental research and veterinary clinical practice, with a focus on investigating disease pathophysiology and evaluating therapeutic strategies.

Particular emphasis is placed on bleomycin (BLM)-induced pulmonary fibrosis in rodent models, including those incorporating aging or genetic modifications, as well as on naturally occurring progressive fibrosing lung diseases in companion dogs and cats. We provide an updated synthesis of the evidence accumulated over the last fifteen years, with particular attention to expert recommendations supporting the complementary use of animal models to address specific research questions.

Importantly, these models provide complementary biological insights and enable the assessment of translationally relevant structural and phenotypic endpoints, including imaging to monitor disease progression and treatment response, and histopathology, both central to human IPF characterization and diagnosis.

To maintain a focused scope, this review does not aim to provide an exhaustive overview of all animal models of IPF-like fibrosing lung disease, or to address comparative aspects beyond imaging and histopathology. To our knowledge, no previous review has comprehensively examined these features within a comparative medicine framework.

## 2. Materials and Methods

### 2.1. Search Strategy

We conducted a comprehensive narrative review of the literature on the most relevant current progress in improving animal models for IPF.

The research project was prospectively (pre)registered in Open Science Framework (OSF) on 28 October 2025 (https://osf.io/7dmv3/overview?view_only=d7585973121243ad93b564faf6525463, accessed on 28 October 2025; DOI https://doi.org/10.17605/OSF.IO/7DMV3).

The search strategy was designed to capture relevant papers investigating the comparative significance of the IPF-like fibrosing lung disease in different species.

We searched the Web of Science Core Collection database for English articles published from January 2005 to August 2025, using the keywords: IPF; idiopathic pulmonary fibrosis; lung fibrosis; pulmonary fibrosis; dog; west highland white terrier; feline; and a combined semantic and Boolean search to obtain broad and relevant results, using the following research questions: “Progress Toward Improving Animal Models for Idiopathic Pulmonary Fibrosis”; “The Histopathology of Idiopathic Pulmonary Fibrosis in dog”; “The Histopathology of Idiopathic Pulmonary Fibrosis in cat”. We have updated our search to identify new articles from the last date of the original search as the start date through 8 April 2026.

From 156 articles, we selected a final set of 40 relevant full-text English research articles, based on the analysis of the title, abstract, and content. Additionally, we searched Google for relevant grey literature to identify updates and additional evidence. This process identified an additional 38 eligible studies, 2 of which were published by June 2026. Reference lists of the selected articles were also screened for relevant studies not identified through the electronic database search, yielding three additional eligible studies [22].

Full-text copies of all the included articles (*n* = 81, published between 1999 and 2026) were retrieved through the WOS database using the National Research Council institutional full-text subscription for further data extraction.

### 2.2. Selection Criteria

For inclusion in this review, journal research articles, editorial materials, book chapters, early access, letters and proceedings papers were considered eligible. Recent reviews were read exclusively for critical appraisal of the authors’ opinions and comparative discussion. In vitro studies and articles in languages other than English were also excluded. Titles, abstracts and full texts were critically examined for relevance, and the following documents were selected:

- Veterinary clinical studies in dogs or cats describing the clinical and/or imaging and/or histological features of spontaneous pulmonary fibrosis.
- Veterinary clinical studies on dogs or cats affected by spontaneous pulmonary fibrosis and subjected to translational therapies.
- Preclinical studies in rodent models of experimental pulmonary fibrosis describing comparative imaging and/or histological features of the disease or potential translational treatments.
- Interspecies comparative pathology reports.

**Figure 1:**
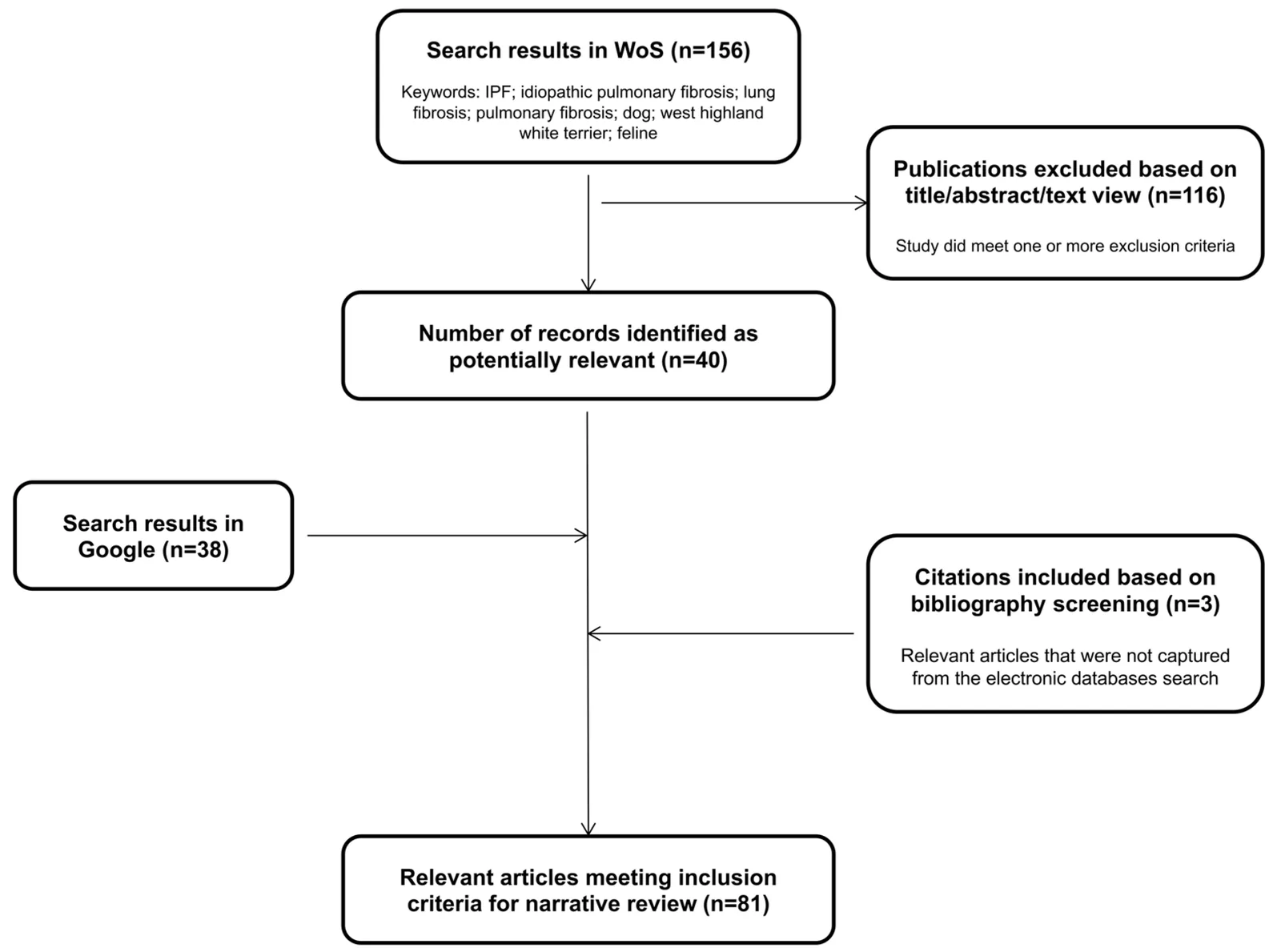
Flow diagram of the study selection process.

This is presented in Figure 1.

## 3. Comparative Interspecies Pathobiology of IPF: State-of-the-Art Experimental and Naturally Occurring Animal Models

### 3.1. Current Human Landscape of IPF

Based on the recent scientific literature cited above, the pathogenetic framework of pulmonary fibrosis involves chronic damage of alveolar epithelial cells, particularly type 2 ones (AECs II), resulting from the complex interplay of environmental factors, comorbidities, and genetic predisposition. Subsequently, AECs II release mediators that promote the migration, proliferation, and differentiation of fibroblasts into activated myofibroblasts, which in turn secrete extracellular matrix (ECM). Epithelial–mesenchymal transition (EMT) may further contribute to this process. As the disease progresses, AECs II become progressively depleted, while macrophages, T and B lymphocytes, and other immune cells exert profibrotic effects, thereby contributing to the progressive lung remodelling and functional deterioration [1].

In particular, so-called “alternatively activated” M2-like macrophages, which exhibit profibrotic and tissue repair functions, appear to play a key role in acute exacerbations of IPF (AE-IPF), characterized by acute respiratory deterioration triggered by idiopathic or infectious insults. These episodes are associated with the appearance of new ground-glass opacities and consolidations superimposed on a UIP pattern on HRCT, along with histopathological features of acute diffuse alveolar damage or organizing pneumonia.

During AE-IPF, M2-like macrophages would release pro-fibrotic and anti-inflammatory mediators, including transforming growth factor-beta (TGF-β), platelet-derived growth factor (PDGF), and CCL18, thereby promoting dysregulated fibroblast proliferation, myofibroblast differentiation, and excessive extracellular matrix deposition [1,3].

Evidence-based guidelines for IPF diagnosis have been developed and periodically updated based on decades of worldwide clinical data [9,10], with recent advances informed by national multicenter registries in the United Kingdom (UK) and the United States of America (USA) [23,24,25].

Overall, current guidelines emphasize a multidisciplinary approach to IPF diagnosis involving clinicians, radiologists, and pathologists, integrating clinical findings with imaging and, in selected cases, histopathology to ensure accurate differential diagnosis. This is essential because IPF symptoms overlap with those of other fibrosing interstitial lung diseases, and radiological and histopathological findings are not always disease-specific.

Differential diagnosis with connective tissue diseases through rheumatologic screening may also be recommended using serological testing [9,10]. On physical examination, IPF is associated with cough, dyspnoea, crackles on lung auscultation and progressive respiratory failure. To date, HRCT remains the reference imaging modality for the noninvasive diagnosis of IPF, enabling the identification of a UIP radiological pattern that correlates strongly with the histological features described above [3], as well as less specific HRCT features, including predominant involvement of the upper, middle, and peribronchovascular lung regions, diffuse ground-glass opacities, nodules, or defined cysts [26].

Advances in radiomics, artificial intelligence (AI), and multimodal imaging, including photon-counting CT, endobronchial optical coherence tomography, PET/CT with [^18^F]FAPI-74 or [^18^F]FDG, and MRI, may improve HRCT-based diagnosis, disease characterization, prognostic assessment, and treatment response evaluation [10,14,15,16,27,28]. These complementary techniques provide structural, functional, and molecular insights into lung tissue mechanics, gas exchange, and inflammatory burden.

Overall, imaging enables noninvasive assessment of the entire lung and supports the early diagnosis of IPF, in accordance with current recommendations [29,30].

When the interpretation of clinical and radiological findings is insufficient to establish a definitive diagnosis, a lung biopsy may be considered [31]. However, its potential benefits and risks must be carefully weighed, considering patient morbidity and the limitations inherent to biopsy, including the small tissue sample obtained and the heterogeneous distribution of IPF lesions [32]. Preliminary studies have explored less invasive approaches, such as bronchoscopic optical coherence tomography (OCT), with promising results [33,34]. Transbronchial lung cryobiopsy (TLCB) is preferred over surgical lung biopsy (SLB) because it is less invasive while providing adequate diagnostic yield, although it requires specialized equipment and expertise. The histological diagnosis of IPF-related UIP is characterized by a heterogeneous distribution of fibroblastic and myofibroblastic foci, which represent sites of “active” lung injury and may be accompanied by a sparse inflammatory cell infiltrate, as well as predominantly subpleural and paraseptal fibrosis.

A defining feature is its “spatial and temporal heterogeneity,” characterized by the coexistence of dense, established collagenous fibrosis, fibroblastic foci, and relatively preserved lung parenchyma. This pattern is consistent with repeated epithelial injury in susceptible individuals [31]. Furthermore, immunohistochemical staining for senescence markers (e.g., p16 or p21) in AECs II often highlights the underlying biological aging process driving the fibrotic response, providing additional diagnostic confidence in atypical cases [35]. Interstitial fibrosis causes architectural distortion of the pulmonary alveoli, and traction bronchiolectasis forms small, honeycomb-like cystic spaces alternating with normal lung areas [9,13,36].

Structural vascular alterations are also observed, characterized by increased vascular density in areas of preserved lung parenchyma and a marked reduction in fibrotic regions. Abnormal angiogenesis and vessel dilation also occur near areas of traction bronchiolectasis and contribute to pulmonary hypertension (PH). These vascular changes involve endothelial–mesenchymal transition (EndMT), similar to that observed in the EMT-mediated alveolar distortion process [36]. These phenomena appear to be driven by inflammatory mediators such as interleukin (IL)-10, tumor necrosis factor (TNF)-α, and nuclear factor kappa B (NF-κB), also acting as profibrotic signals [36].

The Ashcroft scale is an established semi-quantitative method for assessing fibrosis severity in lung histopathological samples from both humans and animal models of IPF. It is based on the visual assessment of progressive thickening of alveolar septa and development of fibrotic tissue. Automated AI-assisted digital pathology may help reduce inter- and intra-observer variability [37,38,39]. Advances in digital pathology, such as fractal histomorphometry, offer promising approaches to improve histological pattern recognition through quantitative assessment of architectural distortion, establish correlations with imaging features, and reduce dependence on biopsy sampling [17].

Accurate diagnosis of IPF is essential to guide appropriate therapeutic management, as early treatment may improve therapeutic outcomes, quality of life, and life expectancy. Unlike other ILDs with well-defined environmental or immune causes, IPF-associated fibrosis cannot be mitigated by eliminating environmental exposures or using immunosuppressive therapies.

Currently approved pharmacological therapies for IPF, including nintedanib, pirfenidone, and nerandomilast, primarily slow disease progression rather than reverse or cure the disease. Consequently, research efforts focus on identifying new therapeutic targets and treatment strategies, with ongoing clinical trials evaluating novel drugs and combination approaches involving antifibrotic and immunomodulatory therapies [7,32,40].

Therapeutic strategies targeting PH and pulmonary vascular remodelling have also been investigated in fibrotic lung disease. In particular, hymecromone reduced pulmonary hyaluronan accumulation, arterial smooth muscle cell proliferation and vascular remodeling in a mouse model of BLM-induced IPF-like disease. It also reduced right ventricular systolic pressure and improved arterial oxygenation [41]. More recently, clinical trials of hymecromone in humans have provided preliminary evidence of functional benefit in patients with ILD-associated PH [8]. Interestingly, hymecromone has also been proposed as a potential treatment for pulmonary fibrosis in dogs, and a clinical recruitment initiative has been launched to identify eligible cases. However, no scientific results have been published to date [42].

Current research into therapeutic options aims to substantially improve the quality of life or survival of patients by acting on specific pathogenic pathways according to the principles of precision medicine [43,44].

However, the limitations of animal models in reproducing human IPF highlight the need for complementary preclinical approaches, combining induced and spontaneous models of fibrotic lung disease, to identify novel pathogenic mechanisms and therapeutic targets. Comparative pathology studies are also essential to account for interspecies differences and ensure the accurate interpretation of research findings.

### 3.2. Overview of IPF Research Using Animal Models: Bridging Induced Rodent Models and Spontaneous Canine and Feline Models

Animal models that closely recapitulate the pathogenetic mechanisms and histopathological features of the human IPF are essential for identifying noninvasive diagnostic and prognostic biomarkers, monitoring disease progression, and developing targeted therapies.

However, no current model fully captures the complexity, heterogeneity, and progressive nature of human IPF.

While sheep and pig models of BLM-induced lung fibrosis offer useful large-animal platforms for studying human-scale lung mechanics and evaluating medical devices and imaging systems, their use is limited by high housing and maintenance costs, limited genetic manipulation tools, and greater ethical and practical constraints [44,45].

Overall, the expert recommendations indicate that murine models of induced fibrotic lung disease are best suited for preclinical translational studies, allowing controlled experimental conditions in a relatively short time frame, genetic manipulation, low cost and ease of management [18]. However, they may not reproduce the chronic evolution of human IPF or the complex role of the exposome in this pathology. Indeed, several compounds have shown efficacy in these animal models, but few have been validated clinically. Interestingly, fibrotic lung diseases can occur spontaneously in several domestic species, including horses [46], donkeys [47] and certain breeds of companion dogs and cats. Experimental models of AE-IPF have also been described in rodents, while spontaneous cases have been reported in companion animals.

Among naturally occurring animal models of IPF-like fibrosing lung disease, dogs and cats may be particularly suitable for complementary translational studies because of both their clinical accessibility and pulmonary anatomy. Unlike other large animal models that bridge the gap between rodents and humans, dogs and cats with ILD are more commonly evaluated and treated in veterinary clinical settings, facilitating case identification and recruitment.

From a comparative perspective, canine and feline models offer anatomical features that may facilitate the study of human respiratory diseases, including IPF. Unlike pigs and sheep, dogs and cats lack a tracheal bronchus arising from the trachea proximal to the main bifurcation. Furthermore, their relatively thin visceral pleura and lack of prominent interlobular septa provide additional anatomical similarities relevant to comparisons with the human lung.

As in humans, dogs and cats also have respiratory bronchioles between the terminal bronchioles and alveolar regions, whereas in pigs and ruminants, the terminal bronchioles predominantly transition into alveolar ducts. Combined with the spontaneous occurrence of fibrosing interstitial lung diseases that share clinical and pathological features with human IPF, these anatomical similarities support the use of dogs and cats as complementary natural models of human pulmonary disease, particularly in studies integrating imaging and histopathology [48,49,50].

Dogs and cats, particularly purebred animals with more homogeneous genetic backgrounds, may serve as valuable comparative models of spontaneous, progressive pulmonary fibrosis. Their longer lifespan than laboratory rodents, shared environmental exposures with humans, and similarities in genetic predisposition, clinical presentation, and disease progression make them relevant models of human IPF [51]. Given the interplay between genetic susceptibility and environmental triggers in IPF, companion animals may also act as sentinels for identifying harmful exposures before they affect humans [4,52].

In developed countries, they have access to advanced veterinary care, including genetic and biochemical screening, HRCT scanning, echocardiography for the assessment of PH, and histological biopsies. This enables the collection of a statistically significant amount of clinical data for comparative research. Moreover, monitoring disease progression in client-owned dogs, including older individuals, provides longitudinal data that can be used to assess treatment responses and support translational studies [50].

Furthermore, drug trials in companion animals with naturally occurring diseases can provide data on clinical outcomes that can be compared across species in a relatively short timeframe. Veterinary recruitment initiatives evaluating human-derived therapies, such as hymecromone or sobetirome, have also been initiated in canine patients, although no published results are currently available [41,53]. In this context, given the lack of specific veterinary therapeutic options, drugs approved for human IPF could be evaluated in naturally occurring disease in companion animals, within a “One Health, One Medicine” framework that may benefit both species while fostering interdisciplinary collaboration [18,50,54].

Overall, combining different animal models according to specific research objectives, could help overcome the limitations of individual models. This approach would also account for interspecies differences in physiology, drug metabolism, and treatment response, potentially improving the success of human clinical trials [49].

Last but not least, beyond the scientific aspects, ethical considerations also support this perspective. The use of naturally occurring IPF-like disease in dogs and cats as a bridging model may contribute to the implementation of the 3Rs principles (Reduction, Replacement, and Refinement) in different ways [55,56].

Scientific knowledge derived from clinical data, in vivo diagnostic information, or histological samples from client-owned animals could help limit the need for experimentally induced animal models in the study of IPF. Furthermore, shifting the perspective of biomedical research from invasive experimental procedures toward veterinary clinical trials may help limit companion animal suffering through the implementation of appropriate standards of care, while also facilitating the translation of improved anesthetic, analgesic, and monitoring protocols laboratory animals.

#### Summary of Data Extraction on the Selected Animal Models of IPF

Data were extracted from studies investigating the selected animal models of IPF to provide a comprehensive overview of their characteristics and applications. For each included study, the main collected information was animal species and breed/strain, sex and age, etiology/experimental protocol, clinical signs related to pulmonary fibrosis, disease progression and prognosis/duration of the study, imaging and histopathological features, molecular and inflammatory markers assessed.

To facilitate comparison, studies were grouped according to the type of comparative model, including naturally occurring canine and feline case reports of pulmonary fibrosis and chemically induced- or genetically modified mouse models of IPF-like fibrosing lung disease. Particular attention was given to critically evaluating the translational relevance of each model by assessing its ability to recapitulate the pathophysiological, imaging, and histopathological features of human IPF.

**Figure 2:**
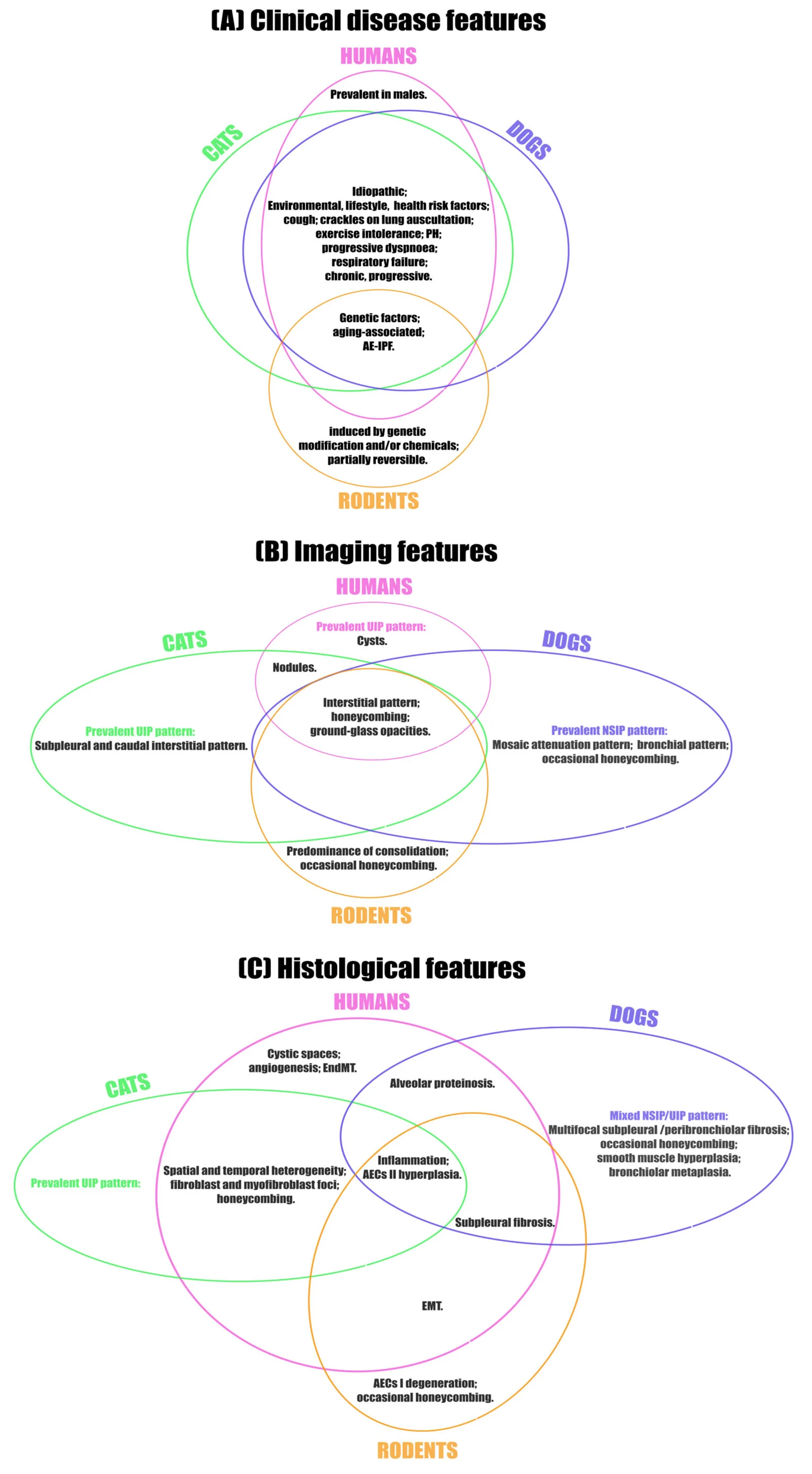
Venn diagram of shared and distinct IPF features across selected animal models and humans. (**A**) clinical disease features; (**B**) imaging features; (**C**) histological features.

The collected evidence was synthesized in a narrative format to compare the strengths and limitations of these animal models, assess their utility in elucidating disease mechanisms and potential therapeutic interventions, and identify current knowledge gaps and future directions for preclinical IPF research. The comparative clinicopathological features summarized in Table 1 and Figure 2 are supported by references in the relevant sections of the manuscript and are discussed in further detail below.

The descriptive analysis of scientific articles identified through our literature search revealed a clear prevalence of experimental studies on rodent models of IPF-like fibrosing lung disease followed by comparative investigations in canine veterinary patients, while case studies on feline pulmonary fibrosis are still limited, despite their potential translational relevance.

**Figure 3:**
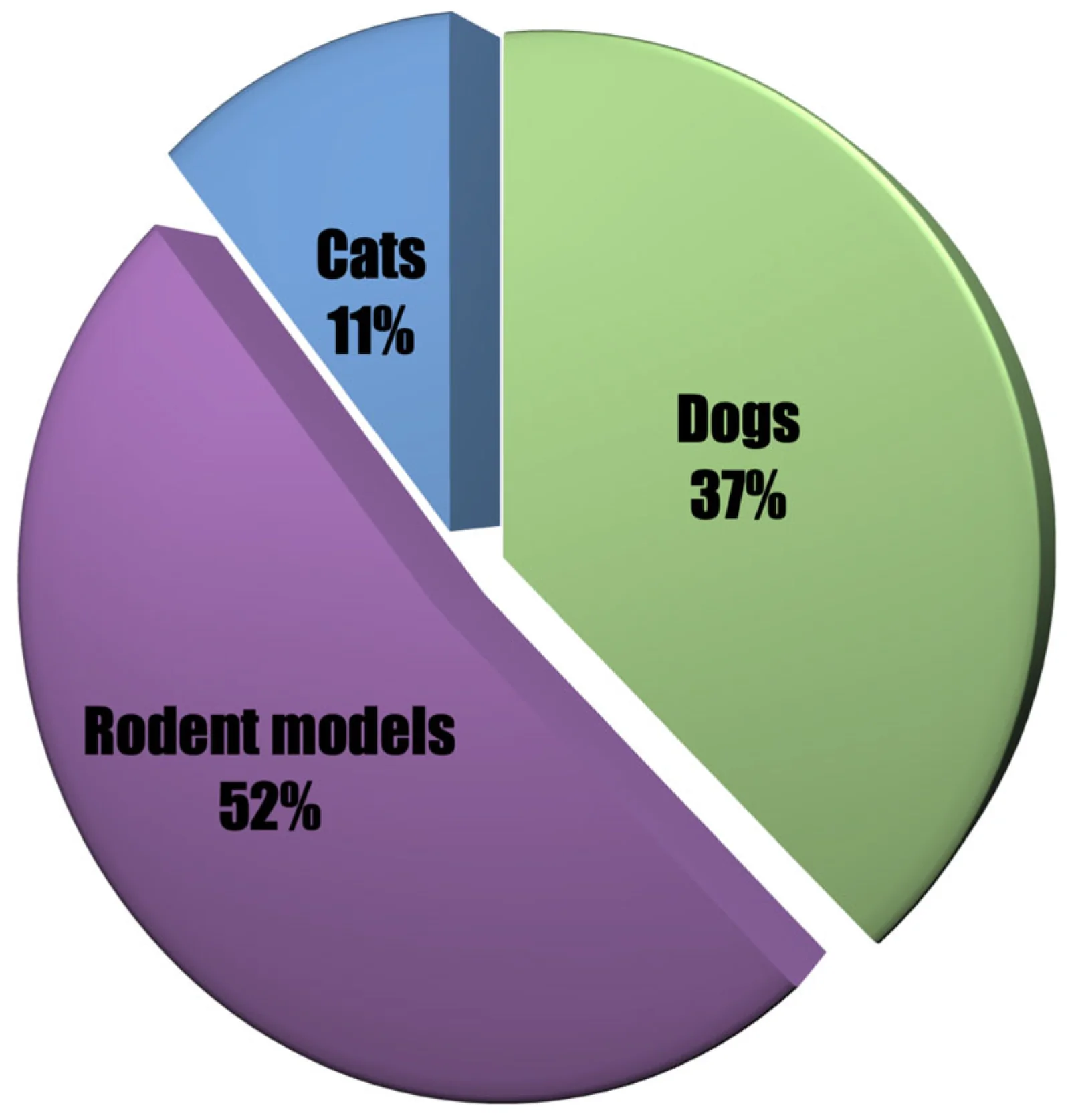
Overview of the scientific literature on the selected animal models of IPF published over the past 25 years. The pie chart illustrates the relative proportion of publications focusing on naturally occurring canine and feline IPF and the principal experimentally induced mouse models, including chemically induced and genetically modified mouse models. Percentages represent the approximate contribution of each animal model to the published literature included in this review.

Figure 3 illustrates the overall scientific production on the selected animal models of IPF from January 2005 to June 2026, expressed as a percentage of the total articles found.

Among the studies on rodent models of IPF-like fibrosing lung disease identified through the literature search, only 14% employed rats, whereas the remaining studies used mouse models. Although rat models offer advantages, such as larger lung size and easier implementation of imaging techniques for repeated assessments of disease progression and treatment response, mice remain predominant in preclinical IPF research. This likely reflects the wider availability of well-established induced and genetically engineered mouse models, as well as the extensive range of molecular and genetic tools.

**Figure 4:**
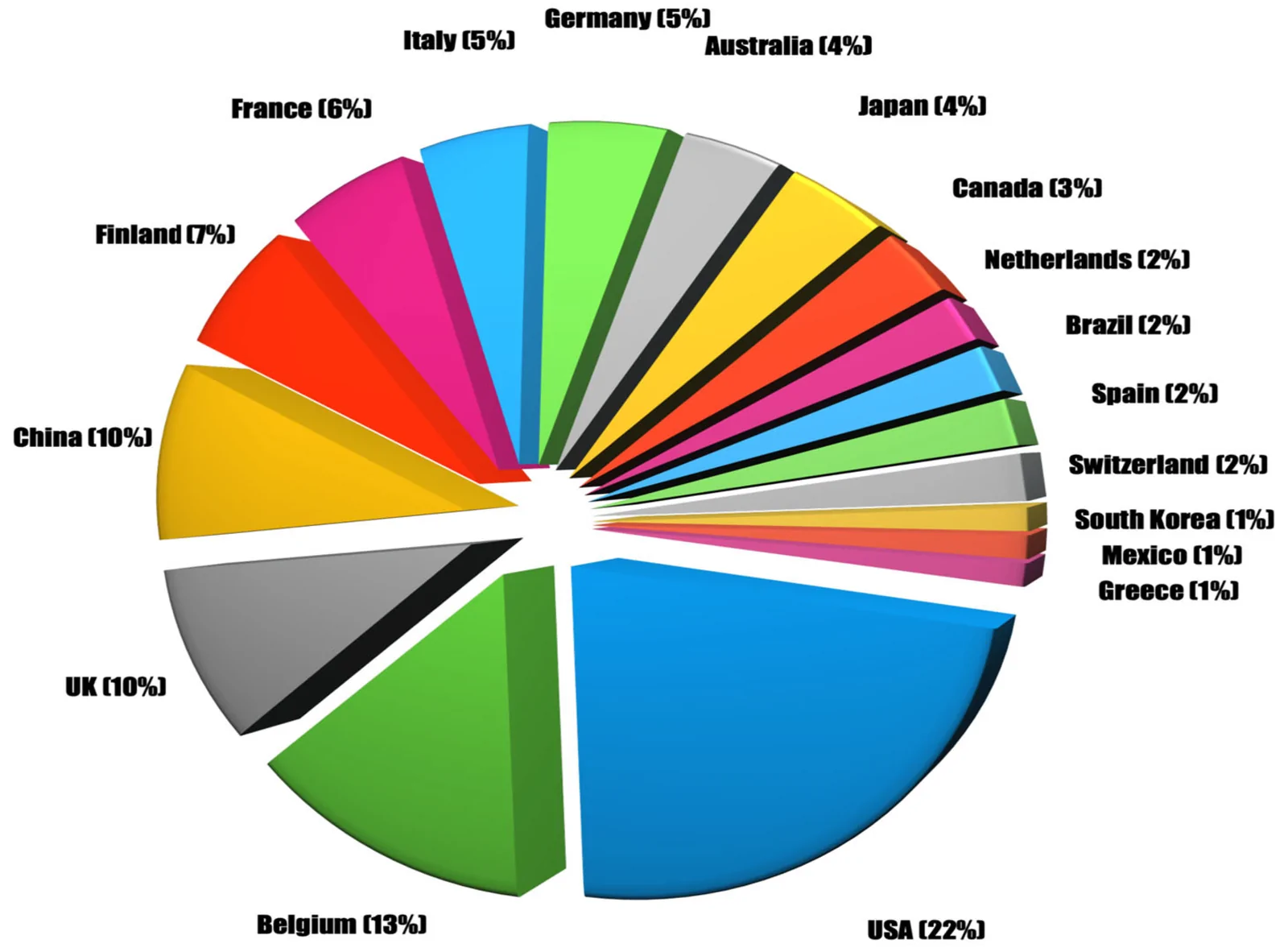
Overview of the scientific production by country.

These characteristics have made mice the preferred species for mechanistic studies of IPF, contributing to the extensive knowledge accumulated in the field. Figure 4 shows the geographical distribution of scientific contributions by country as a percentage of the total number of identified articles. The USA accounted for the largest share of publications on comparative IPF research, followed by several European and Asian countries.

Overall, contributions from Europe, North and South America, Asia, and Australia reflect broad international engagement and widespread interest in advancing knowledge of IPF and developing novel therapies for this progressive, irreversible disease.

**Figure 5:**
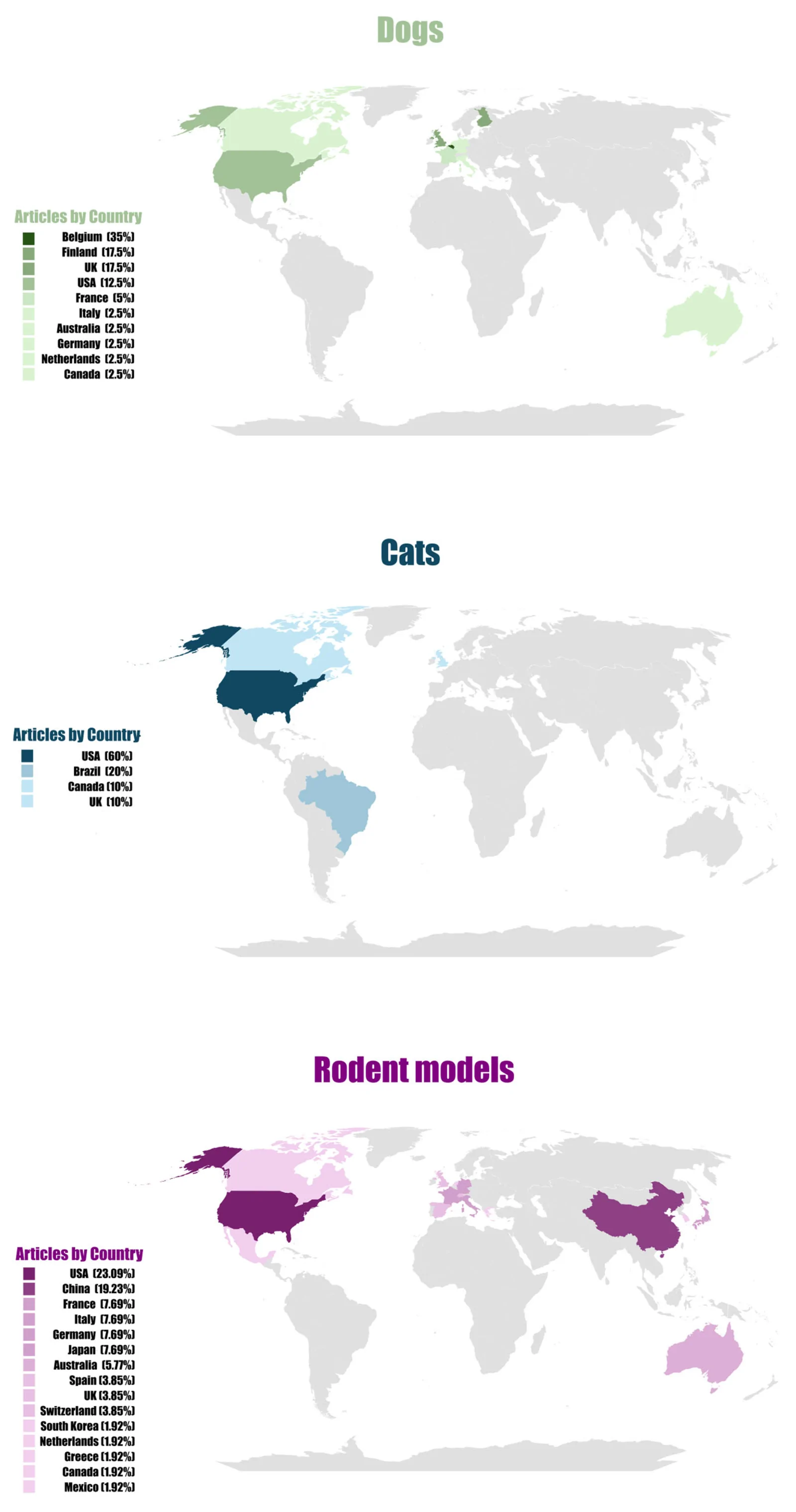
Distribution of the scientific production according to animal model and sorted by country. Maps were created using Microsoft Excel with Bing Maps technology. Data sources: © Australian Bureau of Statistics, GeoNames, Geospatial Data Edit, Microsoft, Navinfo, Open Places, OpenStreetMap, Overture Maps Foundation, TomTom, Wikipedia, Zenrin.

The geographic distribution of publications highlights the growing relevance of comparative approaches and the potential of a “One Health, One Medicine” perspective in IPF research. Figure 5 shows the percentage distribution of publications by species across countries.

Research using rodent models of IPF spans 15 countries across North America, Europe, Asia, and Oceania, but is concentrated in the USA and China. This widespread adoption reflects the importance of conventional animal models for investigating IPF pathogenesis and evaluating novel therapeutic strategies, although scientific output may be influenced by access to specialized animal facilities, advanced preclinical and imaging infrastructure, and financial resources.

In contrast, studies involving the spontaneous canine model of fibrotic lung disease are largely limited to a small number of countries, with Belgium, Finland, and the UK accounting for the majority of publications, followed by the USA and France. The contributions of other European countries, as well as Canada and Australia, remain relatively modest. This limited geographic distribution likely reflects the relatively low number of eligible cases of naturally occurring canine fibrotic lung disease, its association with predisposed breeds, the need for specialized veterinary expertise and referral centers for case identification and monitoring disease over time, and the importance of interdisciplinary research networks.

Similarly, comparative studies employing the spontaneous feline model of fibrotic lung disease have been reported predominantly from the USA and Brazil, with smaller but comparable contributions from the UK and Canada.

The limited number of countries involved in feline pulmonary fibrosis research further highlights the challenges of studying naturally occurring disease, including the low availability of cases, difficulties in establishing a definitive diagnosis, and the lack of coordinated research strategies. Overall, these findings suggest that, while experimental rodent models are widely used internationally, research on spontaneous companion animal models remains concentrated in a limited number of specialized centers. Expanding international collaborations, standardizing veterinary diagnostic criteria based on those used in human medicine, and establishing shared veterinary registries could improve data collection from spontaneous cases and strengthen the translational value of comparative research on IPF within a One Health framework.

**Figure 6:**
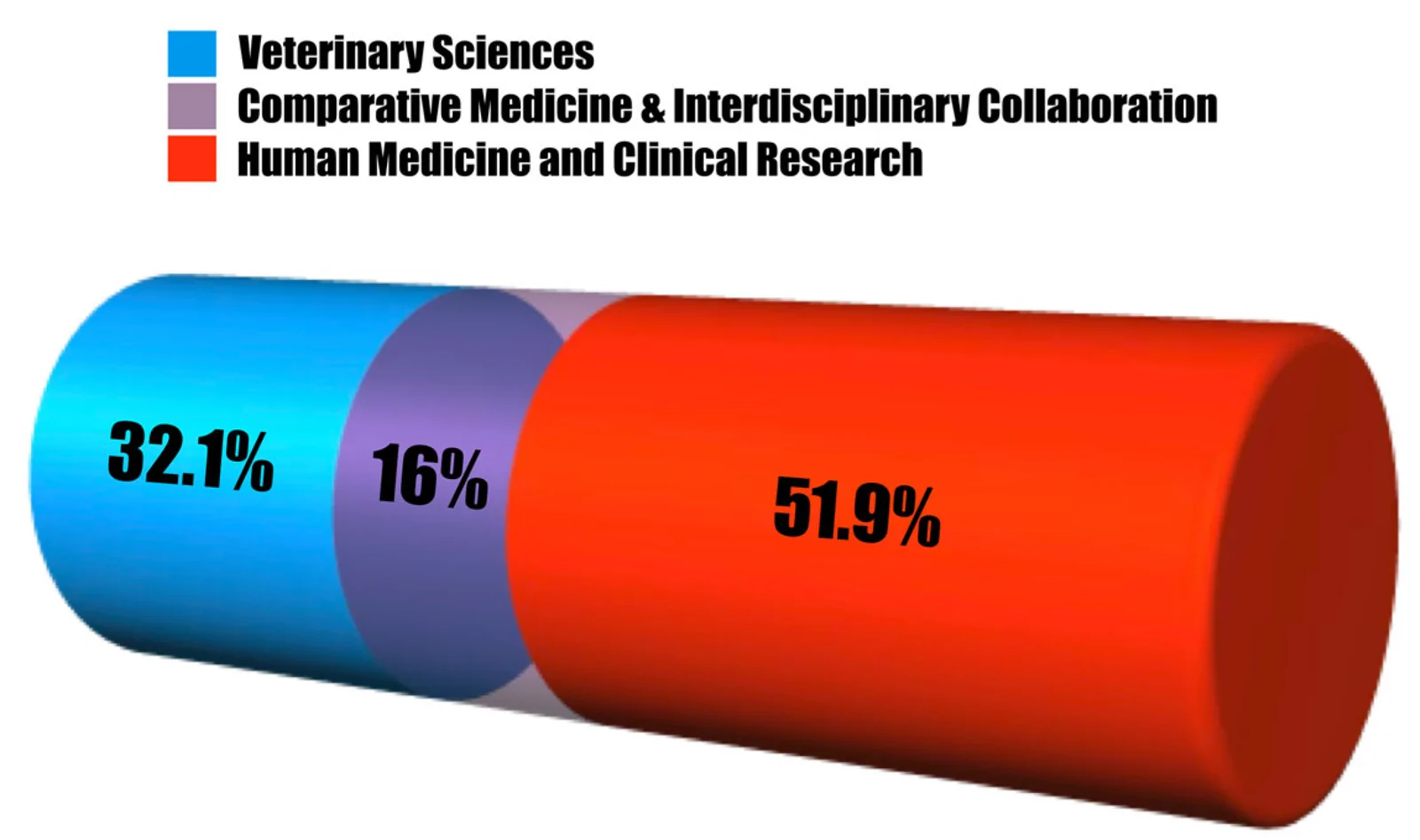
Distribution of the scientific production according to authorship patterns.

As shown in Figure 6, most publications originated from medical research institutions, with substantial contributions also from veterinary clinical and research institutions. The participation of both medical and veterinary institutions highlights the importance of interdisciplinary collaboration and the value of integrating human and veterinary perspectives to advance IPF research and improve our understanding of this complex disease.

### 3.3. Exploring the Potential of Naturally Occurring Veterinary Models of IPF

Advances in small animal veterinary medicine have led to increasingly accurate recognition of IPF-like fibrosing lung disease in both dogs and cats.

The most extensively studied veterinary model of spontaneous IPF is the West Highland White Terrier (WHWT) dog breed, which shares several clinical, histological, and pathophysiological features with human IPF, while also showing species-specific characteristics. Moreover, WHWTs offer the possibility of translational monitoring using advanced imaging techniques.

Spontaneous pulmonary fibrosis is also recognized in domestic cats, sharing with humans a similar anatomy of the distal pulmonary airways and some histological features resembling those of human UIP, including honeycomb structures and fibroblastic foci, as well as cellular alterations involving AECs II and surfactant production.

Comparing the clinical and pathological features of pulmonary fibrosis in humans and companion animals can help identify interspecies differences and clarify the mechanisms underlying spontaneous IPF-like fibrosing lung disease. Notably, pulmonary fibrosis in companion animals can follow a chronic course, more closely resembling the disease progression observed in humans than that typically seen in conventional experimental rodent models of IPF.

These aspects are discussed in greater detail, with supporting evidence, in the following sections.

#### 3.3.1. Updates on Canine Models of IPF

Over the past decade, interdisciplinary meetings and comparative medicine reviews have increasingly discussed the value of naturally occurring IPF in companion dogs, critically evaluating available animal models and identifying priorities for future research. These interdisciplinary efforts aim to provide novel insights into human IPF and contribute to the development of effective therapies for both humans and animals [18,50,51,54,120].

Similar to humans, IPF is often diagnosed in middle-aged or older dogs at advanced stages of the disease, when treatment of the lung lesions is already ineffective, and mean life expectancy is approximately 18 months.

Typical clinical signs observed in dogs with progressive pulmonary fibrosis include dry cough, inspiratory crackles, exercise intolerance, tachypnea or dyspnea and eventually cyanosis, PH and abdominal breathing [85,86,121]. In particular, IPF has been described in terrier dog breeds such as Jack Russell terrier, Staffordshire bull terrier, Scottish terrier and Cairn terrier, and most notably WHWTs, possibly related to a genetic susceptibility to interstitial pulmonary fibrosis disease, with no apparent sex-related differences [51,57,58,59,60,61,62].

Interestingly, a genome-wide association study conducted in WHWTs, based on saliva sample collection by owners, suggested a potential role for the human orthologous gene of polyadenylation factor 7 (CPSF7) in canine IPF. This gene has been implicated in human lung adenocarcinoma, for which IPF would be a risk factor [63].

In addition, genetic variants of the succinate dehydrogenase complex assembly factor 2 (SDHAF2) were found to be associated with IPF in WHWTs [63].

Despite limitations, including potential selection and information biases, this study represents a useful example of a citizen-science initiative that could generate new insights into the pathogenesis of IPF from a “One Health, One Medicine” perspective.

Beyond these genetic findings, comparative studies have identified shared molecular features and potential biomarkers between canine and human IPF.

Gene expression analysis of lung samples from WHWTs affected by IPF-like disease revealed increased expression of genes encoding CCL2, CCL7, CXCL8, CXCL14, IL8, and fibroblast activation protein (FAP), which are involved in inflammation and fibroblast proliferation, compared with clinically healthy WHWTs. These findings were consistent with those reported in human patients and were further supported by immunohistochemistry and serum chemokine measurements.

Furthermore, healthy WHWTs showed higher serum levels of TGF-β1 compared with healthy dogs of other breeds [64,65,66,67].

These findings support the presence of molecular similarities between canine and human IPF, while also suggesting that repeated lung damage due to environmental factors and respiratory pathogens may trigger the disease in genetically susceptible individuals.

For example, chronic pulmonary fibrosis has been described in a young Bull Terrier, possibly triggered by infection with Severe Acute Respiratory Syndrome Coronavirus 2 (SARS-CoV-2), as suggested by positive serological results [68].

Another study in WHWTs investigated the potential role of sensitization to inhaled fungal antigens, which have been identified as a risk factor for chronic pulmonary fibrosis in humans. Notably, Polymerase Chain Reaction of lung samples and serum precipitin assays revealed a higher prevalence of fungal exposure in WHWTs with IPF-like fibrosing lung disease than in healthy controls. These findings suggest that repeated exposure to fungal allergens may contribute to an exacerbated immune response [69].

Several studies, primarily in WHWTs, have characterized the histopathological and imaging features of canine IPF. Since the early 2000s, limited case reports have highlighted similarities and differences between IPF in dogs and humans, describing a mixed histopathological phenotype that shares features with human UIP but also resembles a nonspecific interstitial pneumonia (NSIP) pattern typical of other interstitial lung diseases [62].

Progressive clinical signs of IPF have been associated with increased pulmonary radiopacity in the lung field and marked interstitial or bronchial patterns on thoracic radiographs or HRCT. Some dogs also experience acute exacerbations of respiratory symptoms, similar to human AE-IPF [51,91]. Following treatment failure and death, postmortem histological examination revealed multifocal, regional, or diffuse alveolar septa thickening due to interstitial fibrosis and AECs II hyperplasia, along with chronic interstitial inflammation characterized by lymphocyte and macrophage infiltration, dystrophic mineralization, and perivascular hyalinization. These findings more closely resemble NSIP, another less frequent form of human idiopathic interstitial pneumonia, while other common features of human UIP, such as intra-alveolar fibroblastic foci, subpleural distribution, and honeycombing, have only occasionally been observed [49,57,58,59,60,61,114]. The heterogeneous findings regarding IPF-like disease reported in these studies may be related to differential exposure to various risk factors for UIP, such as cigarette smoking, environmental air pollutants, respiratory viruses, and residential background radiation sources such as radon, which vary by geographic location and housing conditions.

In recent years, increased access of veterinary patients to advanced and specialized care, bronchoscopy and HRCT, as well as intensive palliative and supportive therapies, has improved animal survival and the identification of dogs with more severe lesions at end-stage disease.

Similar to previous studies, eighteen older WHWTs showed diffuse interstitial fibrosis involving the alveolar walls and subpleural or peribronchial areas, associated with inflammation, AECs II hyperplasia and collagen deposition around small vessels, as seen in human NSIP. Interestingly, smooth muscle cell hyperplasia, bronchiolar metaplasia and a honeycomb-like appearance were observed at multiple sites in the most severely affected dogs, predominantly in the caudodorsal lobes, resembling the pattern seen in human UIP [114].

Notably, alveolar proteinosis, a common feature of human IPF, was also identified in these dogs, possibly due to impaired surfactant degradation by alveolar macrophages. However, the typical fibroblast and myofibroblast foci embedded in a myxoid matrix that characterize human UIP were absent. In this context, the N-terminal propeptide of type III procollagen (PIIINP), a marker of fibroblast activity and increased type III collagen turnover, has been investigated in both dogs and humans with IPF [115].

Despite shared pathogenetic mechanisms, interspecific differences in fibrogenic mechanisms, including increased expression of latent TGF-β binding protein (LTBP)-1 and fibrillin-2, may partly explain the distinct histological patterns observed in canine and human IPF. In WHWTs, fibrosis predominantly affects the peribronchial and perivascular regions, whereas in human IPF, it is primarily distributed around the alveoli [65].

Subsequent comparative research demonstrated that activin B, a cytokine belonging to the TGF-β superfamily, was predominantly upregulated in the alveolar epithelium of WHWTs during AE-IPF, probably mediating alveolar damage, whereas activin A appears to be mainly involved in the pathophysiology of human IPF [122]. Furthermore, unlike human patients, no obvious alterations in haemostasis were observed in WHWTs with IPF [123].

Similarly to findings in patients with IPF, including during AE-IPF episodes, and in mouse models of BLM-induced lung fibrosis, a recent study identified distinct populations of profibrotic macrophages and monocytes in bronchoalveolar lavage fluid (BALF) from WHWTs with IPF using single-cell RNA sequencing (scRNA-seq) [124].

These cells may represent potential therapeutic targets and candidate diagnostic biomarkers. Notably, they showed overexpression of osteopontin (SPP1), which is associated with fibroblast proliferation, ECM deposition, and myofibroblast activation mediated by TGF-β1 in humans with IPF. These preliminary findings in canine models further support the potential of SPP1 as a diagnostic biomarker and therapeutic target in IPF [125].

Thoracic HRCT in dogs with suspected IPF supports veterinary diagnostic and prognostic assessment, and promotes a standardized approach comparable to that used in human clinical practice, facilitating comparative research [50].

However, particular attention should be paid to the need for anaesthesia to minimize motion artifacts during imaging in dogs and to echocardiographic signs of PH. Both factors may affect animal welfare, particularly in cases of respiratory failure, and influence HRCT findings [126].

Interestingly, a pilot study demonstrated the feasibility of performing HRCT in awake dogs immobilized with a dedicated veterinary restraint device, avoiding the risk of anesthesia in cases of severe hypoxia [107]. Thoracic ultrasound has also been used as complementary imaging tool, as in human medicine, to investigate thoracic diseases, including IPF, by detecting pleural surface irregularities, ringdown artifacts, and peripheral nodules [108].

The most common radiological features reported in dogs with IPF include diffuse ground-glass attenuation, a mosaic attenuation pattern characterized by alternating hyperattenuated ground-glass areas and hypoattenuated regions of relatively healthy lung or bronchiolectasis, and focal reticular pattern. However, honeycombing and bronchiolectasis, which are crucial in human IPF diagnosis, are only occasionally seen in canine imaging. The extent of lung abnormalities detected by HRCT correlates with clinical severity and is associated with shorter survival [60,91,109].

HRCT angiography in WHWTs with IPF-like disease may facilitate the early detection of PH, a common comorbidity in humans and dogs that is conventionally evaluated by echocardiography and associated with a poorer prognosis [110].

Recently, a pilot study evaluated [^18^F]-FAPI-74 PET/HRCT to assess FAP expression in WHWTs with IPF-like disease, building on evidence of FAP as a biomarker of fibrotic lung diseases and pulmonary adenocarcinoma in both humans and dogs [127].

FAP-targeted PET/CT may offer a noninvasive means of detecting active fibrosis in vivo and monitoring disease progression and treatment response. Further collaborative studies in dogs with IPF could help validate these applications and support the development of improved diagnostic and therapeutic strategies within a “One Medicine, One Health” framework [127].

Overall, canine IPF shares several clinical, histopathological, and imaging findings with human IPF, although important interspecific differences should be acknowledged. Histologically, IPF in WHWTs is characterized by diffuse, relatively mature fibrosis, with features more similar to NSIP than to UIP, although focal subpleural fibrosis and occasional honeycombing may resemble UIP [114]. In contrast, human IPF typically shows a UIP pattern, characterized by patchy subpleural and paraseptal fibrosis, fibroblastic foci, and honeycombing. Similar differences are observed on HRCT. In WHWTs, ground-glass opacity and mosaic attenuation predominate, while reticular changes and occasional honeycombing may also occur. In human IPF, HRCT typically shows basal and subpleural reticulation, traction bronchiectasis, and honeycombing [60,91,109].

Despite these interspecies differences, WHWTs with IPF may complement murine models in studies of human IPF pathogenesis, given the spontaneous onset of the disease and shared environmental risk factors. Although multidisciplinary approaches are increasingly adopted, clinical data, advanced imaging, and histopathological findings have yet to be fully integrated in veterinary medicine. Larger cohorts are therefore needed to expand the available evidence [62].

#### 3.3.2. Updates on Feline Models of IPF

Early reports from the 2000s described domestic cats with histological features resembling human UIP, including interstitial fibrosis, fibroblast and myofibroblast foci, alveolar epithelial and smooth muscle metaplasia, and honeycombing [70].

Impaired surfactant production by AECs II, similar to that reported in familial human IPF, was also observed and associated with pulmonary neoplasms [71,72]. Moderate inflammatory infiltrates, multifocal hyperaemia, and haemorrhages were also reported [70].

Clinical signs overlapping those of human IPF, including cough, altered breathing pattern, lethargy and anorexia have been reported mainly in middle-aged cats, with no apparent breed or sex predisposition. Radiographs have revealed patchy or diffuse interstitial, bronchiolar, and alveolar abnormalities, predominantly in the caudal lung lobes, as well as occasional pulmonary neoplastic nodules.

As with histological lesions, these findings vary in location and extent. No clear toxic, infectious, or parasitic causes have been found, and affected cats have shown poor responses to pharmacological treatment and limited survival [87].

Cats with IPF-like fibrosing lung disease may mask chronic respiratory impairment by progressively reducing their physical activity until an acute event triggers respiratory decompensation. Consequently, clinical deterioration is typically described in the veterinary literature as a rapid or acute progression rather than formally classified as an acute exacerbation, as in human AE-IPF [70,92].

Several case reports have supported these clinical and histological findings, describing HRCT features such as diffuse reticulation, patchy ground-glass attenuation, pleural and bronchial thickening, traction bronchiectasis and multifocal pulmonary consolidations. Echocardiography has also revealed PH and right ventricular dilation and hypertrophy. Similar findings have been reported in humans with IPF, further supporting the comparative value of feline pulmonary fibrosis [88,89,90].

Interestingly, a case study of pulmonary fibrosis in a young cat with clinical symptoms and HRCT features similar to human IPF, but reversible with antimicrobial and anti-inflammatory treatments, further highlighted the importance of a multidisciplinary approach to the differential diagnosis of IPF [111].

Although current clinical guidelines suggest that typical imaging findings may eliminate the need for lung biopsy in suspected IPF, other identifiable and treatable causes of IPF-like changes should be carefully excluded. This is particularly important because these conditions may have a better prognosis, tissue sampling is invasive and costly, and the correlation between HRCT findings and histopathology in veterinary IPF remains poorly established [72,87,91,111].

Overall, feline pulmonary fibrosis shares histological and molecular features with human IPF, suggesting that it may serve as a useful comparative model. However, further studies are needed to clarify the extent of these similarities.

Few publications have investigated feline pulmonary fibrosis to date. This may be partly due to the tendency of cats to mask early clinical signs and to present for veterinary examination only at advanced or terminal stages of the disease. At these stages, even minimally invasive procedures, such as BAL and HRCT, may carry a high anesthetic risk, limiting the feasibility of clinical investigations. Although feline pulmonary fibrosis may represent a useful comparative model for human UIP/IPF, the limited number of available studies calls for caution when interpreting the similarities between the two conditions.

Standardizing diagnostic workflows in veterinary medicine based on human clinical guidelines could improve our understanding of feline IPF and facilitate the translation of findings between veterinary and human medicine [116].

### 3.4. Current Experimental Rodent Models of IPF

Current rodent models of IPF include those induced by BLM, silica, fluorescein isothiocyanate (FITC), or ionizing radiation, as well as genetically modified and aged mouse models, which may be further exposed to exogenous triggers or engrafted with functional human cells. Rodent models of human IPF remain essential for investigating the mechanisms underlying disease progression and for evaluating novel therapeutic approaches. However, their translational relevance has long been a matter of debate because pulmonary fibrosis may resolve in some models, making it difficult to distinguish treatment effects from intrinsic recovery. Others do not fully reproduce the clinical, radiological, and histopathological features of human IPF [73,74].

Also, a relevant conceptual issue is the controversial use of the term “spontaneous” in the preclinical literature. In particular, mouse models of IPF are established primarily through experimental chemical, physical, and/or genetic interventions. Some mouse models are described as developing “spontaneous” pulmonary fibrosis, i.e., without exogenous fibrogenic factors. However, in reality, these mice carry genetic backgrounds that have been experimentally manipulated for purposes unrelated to the induction of fibrotic lung disease or harbour targeted genetic modifications that predispose them to the development of this disease. This inconsistency deserves critical discussion, as it may lead to confusion about what a spontaneous disease model actually represents.

Aged C57BL/6 mice may show increased profibrotic and inflammatory pathway activity and impaired lung repair after injury. They also appear more susceptible to pulmonary fibrosis following additional experimental insults, particularly males [75,76]. Similar results have been reported in nonobese diabetic (NOD) severe combined immunodeficiency (SCID) mice and in several genetically modified, aged mouse models. These include knockout (KO) mice for the Receptor for Advanced Glycation End-products (RAGE) or relaxin (RLX), mice carrying mutations in surfactant protein genes, and models of Heřmanský–Pudlák disease. Compared with younger animals, these mice develop more severe lung inflammation and fibrosis [77,78]. Although these mouse models have been proposed to reproduce age-related spontaneous pulmonary fibrosis phenotype, this interpretation should be treated with caution. It could be argued that, similar to WHWTs, these models possess a genetic background shaped by human intervention, albeit for purposes unrelated to the induction of pulmonary fibrosis. In both cases, fibrosis develops without experimental induction by exogenous insults.

However, despite reproducing several histological and molecular features of IPF, these mice often fail to fully recapitulate the UIP pattern and the chronic progression of the disease in humans.

Furthermore, unlike companion dogs, laboratory mice are kept under controlled environmental conditions and therefore do not capture the multifactorial interactions between genetic susceptibility and environmental exposures that are thought to contribute to IPF development.

Consequently, these mouse models are better suited to studying age-related fibrotic lung remodelling and increased susceptibility to fibrosis than to reproducing spontaneous human IPF.

The widespread use of large animal models is limited by their high costs and operational complexity. Similar constraints affect aged immunodeficient or genetically engineered mice, in which disease development may be prolonged and mortality unpredictable, reducing cost-effectiveness and experimental reliability. As a result, these animals have not become established models of IPF, although they may be valuable for investigating specific pathogenic mechanisms and evaluating targeted therapies.

Similarly, murine models of IPF-like fibrosing lung disease induced by silica, FITC, or ionizing radiation reproduce some features of IPF but generally lack several characteristic histological features of the human disease. Their use is further limited by variability in the fibrotic response, regulatory and operational challenges, and prolonged induction times [74].

Nevertheless, these models can complement other experimental approaches by helping to investigate specific mechanisms of lung fibrosis. For example, a paraquat-induced mouse model of lung fibrosis has been used to study the pivotal role of EMT in IPF progression [79], while a silica-induced mouse model has been used to investigate the role of IL-9 in the pathogenesis and treatment of human IPF [80].

Recent advances in the literature on rodent models of idiopathic pulmonary fibrosis (IPF) have primarily focused on two main groups: bleomycin (BLM)-induced models and models based on aging and/or genetic factors, some of which combine intrinsic susceptibility with additional exogenous stimuli. In light of the focus of this review on the latest evidence regarding imaging and histopathological features of animal models relevant to veterinary clinical practice and experimental research, the following sections will primarily address BLM-induced rodent models [81].

#### 3.4.1. Bleomycin-Induced Rodent Models of IPF: Rationale, Advantages, and Limitations

BLM-induced pulmonary fibrosis was one of the earliest experimental approaches developed to reproduce key features of fibrotic lung remodelling in rodents and has consequently generated a substantial body of evidence on the underlying molecular pathways, cellular responses, and associated imaging and histopathological alterations [18].

In the past, BLM was used as an antineoplastic drug; however, its adverse effects on pulmonary epithelial cells were subsequently recognized. This toxicity has been attributed, at least in part, to the low levels of a specific hydrolase in the lungs, which contribute to the development of pulmonary fibrosis in treated cancer patients. The recognition of these adverse effects provided the rationale for the experimental use of BLM to induce pulmonary fibrosis in animal models, in which it reproduces several key features of IPF, including lung epithelial injury, fibroblasts and myofibroblast activation, and excessive collagen deposition [93,94].

Overall, BLM-induced rodent models are widely used to investigate the mechanisms underlying acute lung injury and pulmonary fibrosis, and to evaluate potential therapies, particularly in mice. In these models, one or multiple doses of BLM trigger an initial inflammatory response, followed by fibrotic remodelling of the lung.

These models offer several advantages, including relative simplicity, cost-effectiveness, and versatility compared with more complex experimental approaches [82]. In addition, they can be readily integrated into multi-hit experimental strategies, including combination with aging and genetic manipulations. Although these approaches entail higher experimental costs, they may better recapitulate the multifactorial pathogenesis of IPF.

The role of sex in rodent models of BLM-induced IPF-like disease is controversial and remains under debate. Early studies using rat models highlighted a greater susceptibility of females to BLM-induced pulmonary fibrosis. More recent studies in mouse models, however, have reported a significant interaction between sex and age, with more severe pulmonary fibrosis observed in older male mice following BLM challenge. Overall, these findings suggest a protective role of estrogens in adult female mice in which pulmonary fibrosis is chemically induced [76,82,83,84].

In this context, a recent preclinical study involving KO mice for nuclear receptor subfamily 4 group A member 1 (NR4A1) and C57BL/6 wild-type mice, in which pulmonary fibrosis was induced by BLM challenge, demonstrated that 17-β-estradiol (E2) upregulates NR4A1 via estrogen receptor α (ERα), in turn suppressing TGF-β-induced lung fibrosis [95]. Overall, in vivo experimental findings were in line with in vitro testing, showing that E2 significantly attenuates TGF-β-induced fibroblast activation, and with ex vivo increased expression of NR4A1 and ERα in lung tissues of IPF patients, suggesting the potential application of E2-based treatments.

Although the severity and persistence of fibrosis vary depending on the species, strain, experimental protocol, and individual response, BLM administration generally induces acute and chronic lung changes, as well as late-stage fibrotic processes, within a relatively predictable timeframe of a few weeks [96]. Comparison of the pathological features of human IPF with those observed in BLM-induced rodent models consistently reveals increased collagen synthesis by myofibroblasts and the production of profibrotic cytokines in both species. However, BLM-induced models also have several limitations, particularly regarding disease progression, including pronounced inflammation, partially reversible fibrosis, and inconsistent changes in pulmonary compliance [97]. Nevertheless, methodological refinements toward minimally invasive and repeated administrations of BLM have improved the ability to reproduce some histological characteristics better reminiscent of human UIP, including AECs II hyperplasia, mild inflammation, fibroblastic foci, and persistent fibrosis in a spatiotemporal heterogeneity pattern [74].

Innovative translational analytical tools improve the characterization of similarities and differences between human IPF and corresponding mouse models. In particular, the reliability and reproducibility of quantitative analyses of pulmonary fibrosis have been improved through in vivo microcomputed tomography (μCT) imaging and histological analysis of murine lung tissue. Fibrosis quantification can be further refined by automated analysis of pulmonary tissue density in digital histological sections stained with Masson’s trichrome and in high-resolution X-ray tomographic 2D images [98,99]. AI-assisted algorithms can map local tissue density by measuring either the proportion of stained collagen matrix across digital partitions of tissue sections or Hounsfield unit (HU) values, using metrics comparable to those employed in clinical settings. These approaches enable precise quantification of collagen deposition across entire lung lobes, reduce inter-observer variability, and show good agreement both between methods and with the conventional Ashcroft scale. Consequently, they may reduce the number of rodent models of IPF required by providing robust quantitative endpoints through serial in vivo imaging.

Recently, a proof-of-concept study using matrix-assisted laser desorption/ionization mass spectrometry imaging (MALDI-MSI) demonstrated substantial molecular overlap while revealing species-specific differences between human IPF and the BLM-induced mouse model. In particular, the ascorbate and aldarate metabolism pathway, as well as chondroitin/dermatan sulphate fragments, showed a high degree of similarity between histological samples from patients with IPF and those from mice treated with 0.5 mg/kg of BLM [100]. These findings are consistent with a previous genomic analysis demonstrating similarities between a rat model of lung fibrosis induced by 1.5 mg/kg of BLM and human IPF lung tissue [101]. However, interindividual variability, protocol-dependent differences, and interlaboratory heterogeneity remain important limitations of the model. Indeed, the severity and distribution pattern of BLM-induced lung lesions may differ depending on the genetic background of the mice, their health status and intestinal microbiome, as well as the batch, dose, route and frequency of BLM administration. Therefore, these experimental parameters should be carefully selected according to the specific objectives of the study [83,102,103,104].

#### 3.4.2. Bleomycin-Induced Rodent Models of IPF: Updated Methods for IPF Induction and Key Histopathological and Imaging Features

Over the past decades, experimental protocols for BLM-induced IPF-like disease in laboratory rodents have been progressively optimized to minimize animal stress and safeguard animal welfare, while refining the timing of in vivo imaging, histological evaluation, and therapeutic intervention. These advances aim to improve the reproducibility and translational relevance of the model, better recapitulate the key imaging and histopathological features of human IPF, and support the reliable preclinical evaluation of novel therapies [81,105].

The administration of BLM directly to the respiratory system at various doses is widely used to investigate specific molecular mechanisms involved in IPF pathogenesis. Following a single administration, the fibrotic response peaks after 2–3 weeks and gradually resolves over the following 6–8 weeks [106].

In particular, an acute inflammatory phase develops approximately 1–7 days after intratracheal (IT) administration of BLM and is characterized by the activation and recruitment of inflammatory cells and the upregulation of pro-inflammatory mediators. This phase is followed by a gradual resolution of the inflammatory infiltrate and increased fibroblast proliferation over the subsequent 7–14 days. A late fibrotic phase is characterized by myofibroblast proliferation and increased deposition of ECM, typically becoming prominent around 21–28 days after BLM administration [82].

Interestingly, a detailed time course analysis of pathological changes in rats treated with 1.5–2.5 U/kg of BLM demonstrated a complex interplay of pro-inflammatory profibrotic cytokines and growth factors, including IL6, IL1β, TNFα, and TGFβ1 during the first 3–7 days after BLM challenge, followed by changes in phospho-Smad (p-Smad) 2/3 and TGFβ receptor I proteins from days 7 to 14 [96]. Notably, the temporal changes in these profibrotic cytokines are considered translationally relevant to human IPF.

However, a single administration of BLM directly into the respiratory system of mice induces pulmonary fibrotic lesions that are predominantly bronchiolocentric with high interindividual variability. Moreover, repeated administration of lower doses of BLM can result in more persistent fibrotic remodelling [82,94].

In this context, minimally invasive methods of BLM administration are highly desirable. Oropharyngeal aspiration (OA) of BLM is recommended as a refinement of IT administration for inducing pulmonary fibrosis in mouse models [18]. When administered twice at the beginning of the experimental period, OA of BLM at a dose of 1 mg/kg produced pulmonary alterations comparable to those induced by IT instillation, while being faster, less invasive, and requiring less equipment and technical expertise.

Notably, it avoids tracheotomy and tracheal intubation, thereby reducing experimental mortality [94]. Furthermore, OA of BLM at 0.8 U/kg may improve reproducibility and ensure a more consistent distribution of lesions in both lungs. This may facilitate the detection of differences in disease severity associated with genetic factors and/or pharmacological treatments, while allowing both lungs to be used for complementary ex vivo analyses. These advantages may, in turn, contribute to further reducing the number of animals required [100]. Interestingly, quantification of fibrosis by in vivo μCT, based on the area of poorly ventilated lung tissue, correlated well with histological findings and hydroxyproline (HYP) levels, enabling in vivo assessment of the effects of antifibrotic drugs [82]. Three IT instillations of 1 mg/kg BLM administered twice weekly to 9-month-old male C57BL/6 mice induced progressive and persistent pulmonary fibrosis for up to 24 weeks after the last administration, closely recapitulating several histological and radiographic features of IPF [117]. Indeed, lung histology showed increased collagen deposition and fibroblast accumulation, along with loss of alveolar epithelial cells type I (AECs I) and AECs II, distal airway involvement, and alveolar remodelling into honeycomb-like cysts. Furthermore, terminal μCT revealed an overall increase in HU values across the lung fields, indicative of enhanced tissue density consistent with fibrosis. Other findings included traction bronchiectasis, subpleural fibrosis, and septal thickening compared to controls.

Recently, morpho-functional changes in IPF modelled by IT (3–5 mg/kg), intraperitoneal (IP; 20–50 mg/kg) and intravenous (IV; 10 mg/kg) BLM administration were evaluated in 20-week-old C57BL/6 mice using in vivo imaging, pulmonary function test (PFT), and ex vivo histological and biochemical analysis [118]. [^18^F]-FDG PET/CT showed higher radiotracer uptake and HU values in the lungs of all BLM-treated mice compared to controls, suggesting an increased fibrotic density and cell metabolic activity, together with a reduction in ventilated lung areas. These findings were consistent with the results of plethysmography, Masson’s trichrome staining, and ELISA-based quantification of plasminogen activator inhibitor-1 (PAI-1) and HYP, especially following IT administration.

In particular, the authors observed that IV BLM administration predominantly induced diffuse subpleural and interstitial fibrosis, whereas IT administration mainly caused heterogeneous peribronchiolar thickening and early inflammation across different lung lobes. These findings suggest that IV BLM may more closely recapitulate human IPF. Overall, transmission electron microscopy (TEM) revealed degeneration of AECs I, hyperplasia of AECs II, and proliferation of interstitial fibroblasts and alveolar macrophages.

BLM-induced acute injury is also useful as a model of AE-IPF to investigate the molecular mechanisms underlying the unpredictable worsening of the disease and to evaluate new drugs for treating these acute episodes in preclinical trials.

Although AE-IPF has traditionally been modelled by combining BLM with a secondary trigger mimicking an infectious stimulus (e.g., respiratory viruses/bacteria or lipopolysaccharides), sequential BLM administration has become the predominant strategy for inducing AE-IPF in recent years [106].

For example, chronic pulmonary fibrosis was induced in male C57BL/6 mice by four doses of 0.8 IU/g of BLM IT every two weeks, followed by two additional administrations of 1.6 IU/g of BLM IT at the same intervals [128]. Overall, in vivo measurements of oxygen saturation (SpO_2_) and lung compliance by plethysmography, together with pulmonary histological and immunohistochemical analyses, showed that the AE-IPF mouse model developed more severe hypoxia and greater impairment of lung compliance than the BLM-induced chronic stable pulmonary fibrosis model without exacerbation. Lung collagen content was also doubled, and the AE-IPF model showed a high mortality rate.

Notably, the AE-IPF mouse model showed additional histological features, including perivascular infiltrates of mononuclear cells, interstitial macrophages and lymphocytes, and intercellular collagen deposits, along with those reminiscent of chronic UIP pattern (subpleural fibrosis, honeycomb cysts, traction bronchiectasis).

Furthermore, increased levels of TNF-α, IL1β, IL6 and hypoxia-inducible factor 1α (HIF1α) were found in the lungs of AE-IPF mice.

Interestingly, the composition of the gut and lung microbiomes plays a significant role in the progression and severity of the acute lung injury occurring early in the pathogenesis of IPF. Susceptibility to BLM-induced IPF-like disease has been associated with a higher prevalence of *Helicobacter* and *Desulfovibrio* in the gut microbiota, increased neutrophil accumulation in the lungs following acute injury, as well as lung microbial dysbiosis and *Klebsiella* proliferation [103,129]. Overall, translocation of commensal bacteria from the gut to the lungs appears to alter the pulmonary microbiome and, consequently, lung metabolite profiles. These changes may, in turn, influence fibrosis progression and affect the severity of BLM-induced weight loss and mortality in mouse models [103,129].

Although ex vivo histology remains the reference method for assessing how well BLM-induced rodent models of IPF reflect human disease, noninvasive approaches, particularly in vivo imaging, are increasingly being incorporated into preclinical studies.

Multimodal imaging allows disease development and progression to be monitored over time in the same animal, thereby reducing experimental variability and the number of animals required.

The primary role of imaging in rodent models is to visualize in vivo disease features comparable to those observed in human IPF, which would otherwise require animal sacrifice and destructive procedures. In this way, preclinical imaging can help optimize disease-induction protocols, identify the optimal timing for administering anti-inflammatory or antifibrotic therapies, and evaluate treatment efficacy before candidate therapies advance to clinical trials in humans.

An example of this application is provided by ultrashort echo time (UTE) MRI, which has been used to characterize BLM-induced pulmonary fibrosis in rodents.

Following oral administration of BLM at doses of 0.1–2.0 mg/kg, depending on species and strain, UTE MRI sequence generated a positive, hyperintense T2* proton signal in the affected lung parenchyma, in contrast to the low-signal, dark appearance of healthy, air-filled lungs. This signal correlated well with histological findings and ex vivo collagen quantification [130].

Among preclinical imaging techniques, μCT has become a relevant translational tool for quantifying disease progression and assessing responses to therapies in rodents.

Recently, serial μCT monitoring has enabled refinement of the BLM administration protocol for inducing pulmonary fibrosis in 18- to 24-month-old male C57BL/6J mice, an age group comparable to 65-year-old patients with IPF, by reducing the regimen to two doses of 2.5 µg/mouse [112].

Based on mean probability distribution histograms of end-expiratory lung density expressed in HU, the authors proposed the use of densitometric and functional ventilation maps derived from μCT to monitor the progression of lung fibrosis and airspace consolidation, which increases X-ray attenuation by replacing air within the alveolar spaces.

They found that increased lung density on in vivo μCT was associated with worsening functional ventilation, higher Ashcroft scores and structural alveolar changes observed ex vivo.

Furthermore, these methodological refinements contributed to improving animal survival and welfare.

In a subsequent proof-of-concept study, mice received nintedanib treatment for two weeks following BLM challenge, providing a reference model for the validation of novel antifibrotic therapies. Longitudinal μCT evaluation was able to reveal less severe lung fibrosis and better pulmonary ventilation in these mice compared to untreated ones, in agreement with histological findings at the experimental endpoint. These results highlight the potential translational value of in vivo μCT imaging for both preclinical and clinical studies [112]. Furthermore, μCT has proven useful to identify the optimal time-window for antifibrotic treatment in BLM-induced mouse models of IPF, that is crucial for drug discovery. The antifibrotic effect of nintedanib, used as a reference treatment, was evaluated in a mouse model of IPF, comparing two treatment windows: days 7–28 and 14–28 after BLM administration [113]. For this purpose, 8-week-old male C57BL/6 mice were challenged with 1 mg/kg BLM administered three times, resulting in lung fibrosis that persisted for up to 28 days. Morphological and functional parameters were assessed noninvasively by μCT at baseline and on days 7, 14, 21, and 28 after the last BLM administration. Lung density maps showed no apparent differences between the two treatment regimens, consistent with the Ashcroft score. Similarly, neither treatment schedule resulted in improvements in lung ventilation maps. In light of these findings, the authors suggested that initiating nintedanib treatment during the inflammatory phase may not provide additional therapeutic benefit and may instead increase animal suffering. Overall, serial in vivo morpho-functional μCT data and ex vivo histological analyses indicated that the most convenient treatment window for nintedanib was between days 14 and 28.

A recent study using [^68^Ga]-FAPI-04 PET/CT monitoring highlighted the need for further research to identify the optimal timing of antifibrotic treatment in BLM-induced mouse models of IPF [131].

This novel radiotracer specifically targets fibroblast-activating protein (FAP), which is overexpressed in active fibrotic lesions. In particular, male C57BL/6 mice received a single IT dose of BLM (5 mg/kg) and underwent serial [^68^Ga]-FAPI-04 PET/CT imaging on days 7, 14, 21, 28 and 35 after IPF induction. The mice exhibited a distinct temporal pattern of radiotracer uptake, characterized by a progressive increase that peaked during the fourth week of disease, followed by a decline in the fifth week. The imaging findings were consistent with ex vivo radiotracer biodistribution and quantitative analysis of FAP immunohistochemistry. Furthermore, the standardized uptake value ratios (SUVRs) between the lungs and other reference tissues showed a decreasing trend following antifibrotic treatment with nintedanib or pirfenidone. Overall, the results highlighted that [^68^Ga]-FAPI-04 PET/CT imaging may represent a useful translational tool for evaluating the progression of pulmonary fibrosis and the response to antifibrotic therapies.

#### 3.4.3. Bleomycin-Induced Rodent Models of IPF: Current Research on Pathogenetic Mechanisms and Emerging Therapeutic Strategies

Despite its limitations, BLM-induced IPF-like disease in rodents remains a useful model for reproducing selected stages of the disease, including epithelial injury, inflammation, fibroblast activation, extracellular matrix remodelling, and fibrosis.

It can therefore facilitate the investigation of pathogenic mechanisms and the identification of potential therapeutic targets, while providing quantifiable parameters for assessing the efficacy of antifibrotic therapies, including the approved drugs pirfenidone and nintedanib [82].

In particular, over the past decade, EMT has emerged as a key pathogenetic mechanism underlying pulmonary fibrosis. This process is characterized by the loss of cell-to-cell adhesion in injured pulmonary epithelial cells, which acquire mesenchymal features as well as profibrotic and proinflammatory properties, resulting in the secretion of various extracellular mediators that promote excessive collagen deposition in the lungs.

Although the mechanisms underlying EMT in pulmonary fibrosis remain incompletely understood, several studies have identified signalling pathways and cellular mediators involved in disease progression in murine models of BLM-induced pulmonary fibrosis [96].

Interestingly, in 8-week-old C57BL/6 mice with IP BLM-induced IPF-like disease, enhanced TGF-β1/Smad2/3 signalling promoted EMT in pleural mesothelial cells, leading to subpleural fibrosis that closely resembles the histopathological pattern observed in human IPF.

Downregulation of miR-18a-5p was implicated in this process, suggesting that this microRNA may represent a potential therapeutic target [132]. The TGF-β1/Smad2/3 signalling pathway also plays a central role in inducing EMT in alveolar epithelial cells following a single IT administration of BLM in C57BL/6 mice. Notably, this pathway has been successfully targeted therapeutically through the IV administration of mouse-induced pluripotent stem cells (iPSCs) 24 h after BLM instillation [133]. Further supporting the therapeutic potential of iPSCs in IPF, a subsequent study demonstrated that IT transplantation of AEC II-like cells differentiated from human iPSCs attenuated lung fibrosis in a rat model when administered 15 days after IT BLM instillation. This effect was associated with inhibition of TGF-β signalling and reduced α-smooth muscle actin (α-SMA) expression [134].

Preclinical evaluation in relevant animal models remains essential for developing new therapies for IPF, with several candidate molecules having shown promising results that warrant further investigation.

Based on evidence that Clara cell secretory protein (CCSP) knockout (KO) mice are particularly susceptible to BLM-induced pulmonary fibrosis, the antifibrotic potential of Antiflammin-1 (AF-1) was investigated [135]. CCSP is predominantly secreted by Clara cells in the respiratory and terminal bronchioles. AF-1 is a peptide that shares a similar C-terminal sequence and biological properties with CCSP.

Adult male C57BL/6 mice received a single intratracheal (IT) administration of BLM (5 mg/kg). AF-1 was then administered intraperitoneally (IP) at 2 mg/kg/day according to three treatment regimens. For disease prevention, AF-1 was administered from day 0 to day 27. For anti-inflammatory treatment, it was administered from day 0 to day 6. For antifibrotic treatment, it was administered from day 11 to day 27.

Preventive treatment with AF-1 (2 mg/kg) significantly attenuated inflammation at day 7 and collagen deposition at day 28, as assessed by histology. However, a clear therapeutic antifibrotic effect was observed only with the higher AF-1 dose of 10 mg/kg.

Furthermore, in vitro experiments have highlighted that the antifibrotic activity of AF-1 is mediated through the CCSP receptor and involve the TGF-β1 signalling pathway.

Consistent with these findings, further evidence has emphasized the potential of peptide-based approaches as therapeutic strategies for IPF. In another study, a 7-amino acid fragment of the caveolin-1 scaffolding domain peptide (CSP7) was shown to attenuate lung fibroblast proliferation and profibrotic activity, as well as AECs apoptosis. In mouse models of BLM-induced fibrotic lung disease, both IP and intranasal (IN) administration of CSP7 reduced lung fibrosis, as assessed by μCT. Histopathological analysis also showed increased p53 expression and reduced TGF-β receptor expression and Smad2/3 signalling [136].

Recently, tissue regeneration strategies have emerged as promising therapeutic approaches for IPF. The therapeutic potential of mouse distal airway stem/progenitor cells (mDASCs) was evaluated in 8-week-old female C57BL/6 mice with BLM-induced pulmonary fibrosis.

The administration of mDASCs by IT instillation 7 days after BLM treatment (3 U/kg) improved survival and pulmonary function, while reducing collagen deposition. These effects were assessed using functional and histopathological assessments, including SpO_2_, Ashcroft score, α-SMA expression, and HYP content. In vitro studies further demonstrated that mDASCs are capable of proliferating and differentiating into AECs I, as evidenced by the expression of specific cellular markers, including podoplanin (PDPN), homeodomain-only protein homeobox (HOPX), and aquaporin-5 (AQP5) [137]. Syngeneic transplantation of AECs II by IT administration in rodent models of BLM-induced IPF-like disease has also been shown to attenuate fibrosis and restore surfactant protein levels. Pulmonary physiology was evaluated in rats receiving 4 U/kg of BLM IT, with AECs II instillation performed at different time points during the first week following induction of pulmonary fibrosis. Lung functional tests, including derecruitability tests at different levels of positive end-expiratory pressure (PEEP) and quasi-static pressure–volume loops, were performed on days 7 and 14 after BLM administration in mechanically ventilated rats. These parameters evaluated the pressures and rates at which alveoli in the injured lungs closed. Interestingly, administration of AECs II three days after induction of pulmonary fibrosis had no significant effect on ventilated alveolar area or lung compliance. Conversely, administration of AECs II seven days after induction of pulmonary fibrosis increased the ventilated alveolar area and improved inspiratory capacity and lung compliance, while attenuating septal wall thickening. These in vivo findings were supported by ex vivo TEM analysis of fixed lung samples, which revealed the presence of active forms of surfactant in treated rats compared with untreated controls, suggesting recovery from lung injury during the pro-fibrotic phase [138].

Nerandomilast, a preferential inhibitor of phosphodiesterase 4B (PDE4B), has recently been approved as a new therapeutic option for human IPF, following preclinical studies in rodent models of BLM-induced disease [7,139]. In particular, these animal models have helped elucidate the mechanisms of action of this drug and its target signalling pathways. Rats treated with 1 mg/kg BLM via IT administration and nerandomilast by oral gavage showed improved pulmonary function in vivo, reduced lung density on μCT and lower Ashcroft scores compared with untreated controls. Furthermore, nerandomilast effectively reversed the dysregulation of several genes and signalling pathways implicated in both human IPF and BLM-induced IPF-like disease in rats, including cyclic adenosine monophosphate (cAMP), G-protein coupled receptor (GPCR), cAMP response element binding (CREB) signalling, and pathways involving Matrix Metalloproteinase-7 (MMP7), IL4, TGF-β 1, and TNF. In vitro experiments using primary human small airway epithelial cells (SAECs) further supported the anti-inflammatory and antifibrotic mechanisms of action of nerandomilast [6].

Overall, current preclinical research highlights that methodological limitations in assessing the efficacy of treatments for IPF represent an additional challenge, alongside the inherent limitations of rodent models of IPF.

Combining emerging technologies, such as preclinical imaging and immunohistochemical detection of degraded collagen, with traditional methods at appropriate time points could improve the assessment of pulmonary fibrosis progression, particularly in advanced disease stages, and facilitate the development and evaluation of novel antifibrotic therapies [140].

Further improvements may come from assessing functional and molecular parameters measured in IPF clinical trials, such as forced vital capacity (FVC), diffusing capacity for carbon monoxide (DLCO), and the biomarker MMP-7, and from applying AI to enhance in vivo imaging and histological quantification of pulmonary fibrosis. Integrating these clinically relevant endpoints could ultimately improve the translational relevance of preclinical IPF models [141,142].

#### 3.4.4. Aging- and/or Genetically Driven Rodent Models of IPF

Although BLM-induced rodent models of IPF have advanced our understanding of fibrotic lung injury, they do not fully reproduce the chronic course of human IPF.

Mouse models that develop irreversible pulmonary fibrosis with aging may complement these models by providing further insights into the mechanisms underlying the transition from acute injury and inflammation to persistent, progressive lung fibrosis. In particular, they may better reproduce key features of progressive pulmonary fibrosis, including cellular senescence, impaired tissue repair, ECM accumulation, and altered responses by epithelial, mesenchymal and immune cells. These models may also provide a clinically relevant platform for evaluating therapies aimed at slowing or reversing disease progression.

Some studies have used aged mice to investigate the delayed resolution of BLM-induced pulmonary fibrosis. In female 52–56-week-old Tg(Acta2-CreERT2)/+; tdTomatoflox mice, treated with 2 U/kg of BLM IT and subsequently with tamoxifen IP, αSMA-expressing myofibroblasts were traced by bright red fluorescence. Resident mesenchymal cells (rMCs) were isolated from these mice and from 8-week-old C57BL/6 mice, which served as young controls. Alveolospheres assays demonstrated that rMCs isolated from aged mice had impaired stem cell-supporting activity for AECs II. Furthermore, BLM-treated aged mice showed increased and persistent pulmonary fibrosis compared with young mice, as assessed by the Ashcroft score. Interestingly, the increased susceptibility of aged mice to fibrosis was associated with the prevalence of Collagen Triple Helix Repeat Containing 1-positive (Cthrc1^+^) activated myofibroblasts (aMYFs), a subpopulation of myofibroblasts involved in wound healing and tissue remodelling. Cthrc1^+^ aMYFs have been implicated in the pathogenesis of fibrotic diseases, including IPF, through their excessive production of ECM components, particularly collagen. These findings suggest that age-related alterations in myofibroblast populations may contribute to the persistence of pulmonary fibrosis and identify Cthrc1^+^ aMYFs as a potential therapeutic target for IPF [119].

In D1CC^+/+^ × D1BC^+/+^ transgenic mice, a model of rheumatoid arthritis and pulmonary fibrosis on DBA/1J-background, IT administration of BLM followed by microbubble-assisted sonoporation resulted in a distinct temporal pattern of lung disease. The model was characterized by an acute phase resembling NSIP, followed by a chronic fibrotic phase with a honeycomb-like appearance reminiscent of UIP. These pathological alterations persisted for up to 14 weeks after BLM administration, as assessed by Masson’s trichrome staining at different experimental time points [143]. This model could therefore provide a useful platform for investigating the mechanisms underlying the onset of NSIP and its progression toward UIP-like features, and for evaluating therapeutic interventions targeting both the acute and chronic phases of IPF.

Current research aims to refine IPF models to better predict the efficacy of new therapies in humans and support the development of treatments that not only slow disease progression but also halt or reduce IPF-related pulmonary remodelling.

Therapeutic strategies based on antibodies that specifically target epitopes of human antigens, as well as immunosuppressive or immunomodulatory molecules and cell-based therapies, cannot be adequately evaluated in conventional animal models because of interspecies differences in receptors and cytokines, thereby limiting the assessment of their efficacy [144]. In this context, the literature has recently highlighted the potential advantages and limitations of humanized mouse models targeting human-specific pathways.

Humanized immunocompromised mouse models of IPF are valuable for investigating the profibrotic mechanisms driven by human cells in vivo and evaluating the therapeutic efficacy of potential antifibrotic agents. These models have been developed by IV injection of cells isolated from lung biopsies or explants from patients with IPF into severe combined immunodeficiency/beige (SCID/bg) or NOD/SCID IL2Rγ^−^/^−^ (NSG) mice. A single cell type (cultured fibroblasts) or mixed cell types (immune, epithelial, stromal, endothelial, pericytes, and stem cells) were injected IV in SCID/bg and NSG mice, respectively, to induce persistent lung remodelling. Notably, NSG mice show increased engraftment of several human cell types because they lack functional natural killer cells, natural killer T cells, and other innate immune cell types, making them useful for exploring the role of both non-immune and immune cells in human IPF. The mice underwent therapeutic interventions and were subsequently sacrificed at different time points for histological and biochemical analyses.

The IV injection of cultured fibroblasts from patients with IPF, but not those from normal lung tissue, induced lung remodelling in SCID/bg mice, as demonstrated by a significant increase in lung HYP content.

Furthermore, treatment with IL-13-phosphatidylethanolamine (PE), which targets fibroblasts expressing IL-4 and IL-13 receptors, markedly reduced interstitial pulmonary remodelling in SCID/bg mice compared with saline-treated controls, as demonstrated by Masson’s trichrome staining and a significant reduction in HYP content. Similarly, IV injection of mixed cell suspensions derived from lung explants of IPF patients induced marked subpleural and interstitial remodelling in NSG mice. Interestingly, given the heterogeneity of histopathological patterns and cellular populations within lungs of IPF patients, the phenotypes and therapeutic response observed in these humanized models were variable. Therefore, the authors suggested paying attention to some methodological issues, including the selection of patient-derived cells used in preclinical studies before testing specific therapies, especially the proliferation/senescence status of fibroblasts from IPF patients before their injection into immunocompromised mice. Overall, therapeutic administration of AMD3100, a C-X-C chemokine receptor type 4 (CXCR4)-specific inhibitor, attenuated lung remodelling in NSG mice, as demonstrated by Masson’s trichrome staining and HYP quantification [145].

In humanized mouse models used to study human immune responses in IPF, a major challenge is graft-versus-host disease (GvHD), characterized by an immune reaction of transplanted human immune cells against foreign mouse tissues. This experimental complication reduces the timeframe for studies and increases animal mortality, thus hindering long-term studies [146]. In light of this, a promising humanized mouse model of IPF was recently developed by intrahepatic injection of human hematopoietic stem cells (HSCs) into 4-day-old, irradiated NOD.Cg-PrkdcscidIL2γgtm1 Sug/JicKoat (NOG) mice, followed by IPF induction by administration of OA BLM in adulthood. Ex vivo μCT and histological findings confirmed the engraftment of human cells in the lungs and BALF of these mice. Notably, this model recapitulated the pathophysiological progression of IPF observed in humans and showed attenuation of fibrosis following nintedanib treatment. Furthermore, the BLM-induced, HSC-humanized IPF mouse model did not develop GVHD for 11 weeks, until the end of the experimental period [147]. This model therefore represents a valuable tool for investigating human immune responses and for the preclinical evaluation of immune-targeted therapies in human IPF.

Genetic modification of key cellular and molecular pathways, together with the introduction of their human counterparts, may represent an alternative strategy for further humanizing the lung microenvironment. For example, mouse models of IPF carrying mutations of Surfactant Protein C (SFTPC) gene identified in human patients could reproduce the dysfunction of AECs II, considered an early and pivotal event in the pathogenesis of IPF. In this context, a chronic Sftpc^C121G^ fibrosis mouse model was developed in which expression of the Sftpc mutation can be induced by tamoxifen (TMX). This model developed progressive pulmonary fibrosis as a consequence of AECs II dysfunction, without the need for exogenous lung injury. It shared several features with human IPF, including its clinical course, physiological impairment, alterations in parenchymal cellular composition, and fibrosis-associated biomarkers. Noteworthy, TMX induction in 48–52-week-old Sftpc^C121G^ mice resulted in more pronounced pulmonary fibrosis compared with younger mice, suggesting an age-related susceptibility to fibrosis [148]. Therefore, this model could represent a significant methodological advance for preclinical studies and antifibrotic drug discovery. Another recently described mouse model, the Ifngr1^−^/^−^Rag2^−^/^−^ mouse, developed progressive and irreversible pulmonary fibrosis during aging in the absence of exogenous triggers. This genetically modified mouse model lacks the acquired immune system due to the deletion of the Rag2 gene and is also deficient in interferon (IFN) γ signalling, a critical suppressive pathway for group 2 innate lymphoid cells (ILC2s). In particular, ILC2s are activated by endogenous factors, including cytokines, lipids, and neuropeptides, and in vitro studies have demonstrated that they can promote collagen production by fibroblasts.

Between 10 and 20 weeks of age, these mice developed an inflammatory phase characterized by increased inflammatory cell counts in the BALF and mild-to-moderate lung collagen accumulation. After 20 weeks, pulmonary fibrosis progressively worsened.

Although aged Rag2^−^/^−^ mice also develop IPF-like changes without external stimuli, 22-week-old Ifngr1^−^/^−^ Rag2^−^/^−^ mice showed more severe pulmonary fibrosis than age-matched single-KO mice, as assessed by μCT and histological analyses.

Notably, the number of ILC2s was significantly increased in fibrotic areas, suggesting a link between ILC2 accumulation and pulmonary fibrosis.

In contrast, pulmonary fibrosis was not observed in Ifngr1^−^/^−^Il2rg^−^/^−^Rag2^−^/^−^ mice, in which ILCs are depleted as a consequence of impaired IL-2Rγ signalling. Interestingly, increased numbers of ILC2 have been observed in the BALF of patients with IPF, along with increased expression of IL-5, IL-13, and IL-1 receptor-like 1 (IL1RL1) in ILC2s, suggesting their activation. Overall, these preclinical results suggested that loss of IFN-γ signalling could promote ILC2s activation during the inflammatory phase of IPF, thereby stimulating collagen production by fibroblasts. Overall, such findings highlight the potential translational value of Ifngr1^−^/^−^Rag2^−^/^−^ mice as a model for investigating the role of ILC2s in IPF pathogenesis [149].

## 4. Comparative Considerations and Translational Perspective: Complementary Applications of Available IPF Models

Given the idiopathic nature and complex, multifactorial pathogenesis of IPF, no animal model can fully recapitulate the disease. Therefore, it is important to identify which pathological features of human IPF are reproduced by each model, and to recognize its limitations. Selecting an animal model based on the specific research question is essential for the appropriate interpretation of findings from biomedical studies.

Experimental mouse models remain indispensable for mechanistic studies because they are reproducible and allow flexible experimental designs. However, they cannot fully reproduce the chronic, progressive, and heterogeneous nature of human IPF. This limitation has led to growing interest in naturally occurring fibrotic lung diseases in companion animals, particularly dogs and cats.

These animals share long-term environmental exposures with humans and show important similarities to human disease in both pathology and imaging findings. However, their use in comparative research is limited by several factors, including high costs, the need for close collaboration between veterinary and clinical medicine, differences in standard-of-care practices, and diagnostic challenges.

BLM-induced models in immunocompetent rodents are widely used to study IPF-related mechanisms and develop diagnostic and therapeutic strategies. Compared with more complex mouse models, they are also easier to use and more cost-effective.

However, as clinical care increasingly relies on targeted and personalized treatments, concerns have been raised about the ability of these models to predict the efficacy of new therapies in humans.

In this context, aged and genetically modified mouse models may better reproduce specific pathogenic mechanisms and aspects of the multifactorial etiology of human IPF. Humanized mouse models, in turn, may provide an experimental system that incorporates cellular and molecular components of the human lung microenvironment. However, mouse models carrying single genetic mutations generally fail to fully recapitulate the pathophysiology and phenotype of human IPF. Moreover, both genetically modified and humanized mouse models retain pulmonary parenchymal cell types of murine origin, which play key roles in ECM deposition, EMT, and fibroblast activation. thereby introducing interspecies biases that may limit their translational relevance. These interspecies differences may introduce biases that limit the translational relevance of these models.

These limitations could potentially be addressed through future methodological refinements, including the co-transplantation of patient-derived airway or alveolar organoids and the generation of transgenic mice expressing human metabolic pathways. Furthermore, the timing and mode of interventions targeting specific molecular pathways should be carefully considered to ensure the validity and interpretability of the resulting readouts. In this respect, histopathological analyses have historically played an essential role in comparative studies by identifying similarities between human diseases and their corresponding animal models. However, the integration of novel translational techniques and biomarkers into the research framework, including advanced preclinical imaging and functional assays, is increasingly recommended to improve the translatability of drug discovery. Furthermore, histological and imaging endpoints should be carefully selected and harmonized with clinical protocols, and their interpretation should account for the strengths and limitations of each model. Table 2 provides an overview of the translational potential and main limitations of the selected animal models of human IPF, integrating the evidence and key considerations discussed throughout the relevant sections of the manuscript.

Overall, combining the mechanistic insights provided by mouse models with the naturally occurring features of canine and feline disease, alongside conventional 2D cell cultures and advanced 3D systems, such as lung organoids, co-culture models, and lung-on-chip platforms, may provide a more comprehensive understanding of IPF pathogenesis and support the evaluation of new therapeutic strategies. Achieving this goal will require closer collaboration among researchers from different disciplines and sustained joint research efforts.

## 5. Concluding Remarks

IPF remains a fatal lung disease, whose pathogenesis is still incompletely understood and for which therapeutic options remain limited. Currently approved drugs, including nintedanib, pirfenidone, and nerandomilast, target key signaling pathways involved in fibrogenesis and can slow disease progression, although their benefits remain limited. Lung transplantation remains the only potentially curative treatment. A major challenge in developing new therapies is the lack of experimental models that adequately reproduce the key features of human IPF. Animal models are essential for investigating the complex interactions among genetic, biochemical, and environmental factors that contribute to disease development, whereas in vitro systems are particularly useful for studying specific cellular and molecular mechanisms. However, no consensus has yet been reached on the most suitable animal model for IPF research.

A paradigm shift in the selection of animal models for IPF is urgently needed. Rather than relying on established practices or model availability, selection should be guided by practical feasibility, predictive validity, and the ability of different models to complement one another.

Achieving this goal requires strong collaboration among human and veterinary clinicians, imaging specialists, pathologists, and basic and translational researchers.

Although establishing such collaborations requires time and resources, it could promote a more comprehensive understanding of IPF and support the development of more effective therapies. This approach builds on the multidisciplinary framework already used in clinical practice, where IPF diagnosis relies on the integration of clinical, laboratory, imaging, and, when necessary, histological findings.

Promoting interdisciplinary collaborations at both national and international levels, alongside the development of open-access databases and open-science frameworks, could facilitate data sharing and foster a more comprehensive understanding of the comparative pathology of IPF.

Although murine models have been essential to IPF research, other animal models may help address specific aspects of disease pathogenesis and improve the translational relevance of preclinical findings. Advances in veterinary medicine and technology may further support this approach. In this context, clinical trials in companion animals could help bridge the gap between experimental animal research and human medicine while providing companion animals with access to advanced veterinary care.

One Health funding strategies could support collaboration among medical and veterinary academies, hospitals, research laboratories and pharmaceutical companies. These partnerships could facilitate multicenter veterinary clinical trials, promote standardized research methodologies and harmonized diagnostic approaches across biomedical disciplines, and support the identification of molecular and imaging biomarkers shared across species. Together, these efforts could advance both human and veterinary medicine in line with the “One Medicine, One Health” framework in biomedical research.

Finally, integrating naturally occurring companion animal models with conventional experimental approaches could support the 3Rs principles by reducing the number of laboratory animals required, providing complementary evidence that may replace some experimental approaches where appropriate, and refining research practices by minimizing animal suffering and improving standards of care.

Interestingly, the issue of “unrecognised 3Rs” has been acknowledged, although it has not been formally defined. This term refers to sources of complementary knowledge whose contribution to the 3Rs goes unnoticed, either because they arise from professional activities not specifically designed to develop or implement a related method, or because their contribution is not explicitly reported in the resulting publications. A One Health approach, which integrates human, animal, and environmental health, could help identify otherwise overlooked opportunities to partially replace animal experimentation with veterinary epidemiological data and clinical cases, as discussed above [150,151].

Ultimately, applying the 3Rs principles to IPF research may not only improve animal welfare but also generate high-quality, reproducible, and reliable data with translational relevance for both human and animal patients.

## Figures and Tables

**Table 1:** Comparative pathology of IPF across species.

Disease Feature	Humans (Reference)	Dogs	Cats	Rodent Models
Etiology	Idiopathic origin; aging-associated; more prevalent in males; multifactorial triggers: genetic predisposition, environmental/ lifestyle risk factors, and comorbidities [1,2,3,4,5].	Idiopathic origin; aging-associated; high prevalence in WHWTs; hereditary susceptibility also in other terrier breeds and family lines; no sex predisposition; naturally occurring; multifactorial triggers: genetic predisposition, environmental risk factors, and comorbidities [51,57,58,59,60,61,62,63,64,65,66,67,68,69].	Idiopathic origin; aging-associated; sporadic, natural occurrence with no sex or breed predisposition; multifactorial triggers: genetic predisposition, environmental risk factors, and comorbidities [70,71,72].	Pulmonary fibrosis induced by targeted genetic modification and/or chemicals administration (bleomycin, silica, radiation); aging-associated susceptibility in some mouse strains/genetically modified mice; controversial role of gender in rodent species [73,74,75,76,77,78,79,80,81,82,83,84].
Clinical signs	Cough; crackles on lung auscultation; exercise intolerance; PH; progressive dyspnoea and respiratory failure [9,10].	Cough; crackles on lung auscultation; exercise intolerance; tachypnoea; cyanosis; PH; progressive dyspnoea and respiratory failure [85,86].	Cough; tachypnoea, respiratory distress; open-mouth breathing; PH; lethargy/anorexia; progressive dyspnoea and respiratory failure. [87,88,89,90].	Variable; respiratory signs often subtle unless severe [73,74].
Disease progression/ prognosis	Chronic, progressive, occasional acute exacerbations, irreversible; poor prognosis [1,3,9,10].	Chronic, progressive, occasional acute exacerbations, irreversible; poor prognosis [51,85,86,91].	Features of chronic disease with acute and rapid clinical worsening, irreversible; poor prognosis [70,87,88,89,90,92].	Chemical models: acute, partially reversible; inducible acute exacerbations; genetic models: progressive epithelial cell dysfunction, persistent fibrosis [73,74,75,76,77,78,79,80,81,82,83,84,93,94,95,96,97,98,99,100,101,102,103,104,105,106].
Imaging (primarily HRCT)	Prevalent UIP pattern: subpleural and lower interstitial pattern; honeycombing (traction bronchiectasis); ground-glass opacities, nodules, cysts [1,3,9,26].	Prevalent NSIP pattern: diffuse increased lung radiopacity; mosaic attenuation pattern; interstitial or bronchial pattern; ground-glass opacities; occasional honeycombing [50,51,91,107,108,109,110].	Prevalent UIP pattern: subpleural and caudal interstitial pattern; ground-glass opacities; honeycombing (traction bronchiectasis); nodules [72,87,88,89,90,111].	Imaging findings are reminiscent of UIP pattern, but rarely fully recapitulate human HRCT; mainly experimental μCT; ground-glass opacities; airspace consolidation; interstitial pattern; bronchiectasis; occasional honeycombing [112,113].
Histopathology	Prevalent UIP pattern: spatial and temporal heterogeneity; fibroblast and myofibroblast foci; honeycombing (traction bronchiectasis); cystic spaces; moderate inflammatory infiltrate; AECs II hyperplasia; abnormal vascular density and angiogenesis; EndMT; subpleural fibrosis; EMT; alveolar proteinosis [9,10,11,30,31,35,36].	Prevalent NSIP pattern: diffuse interstitial mature fibrosis; common perivascular concentric fibrosis and inflammation; AECs II hyperplasia; multifocal subpleural/peribronchiolar fibrosis, occasional honeycombing and AECs II hyperplasia, more closely resembling UIP, with alveolar proteinosis in severely fibrotic lung areas; more severe lesions in caudal lobes (smooth muscle hyperplasia, bronchiolar metaplasia, honeycombing, intra-alveolar fibrosis) [49,57,58,59,60,61,62,65,114,115].	Prevalent UIP pattern: spatial and temporal heterogeneity, interstitial fibrosis; fibroblasts and myofibroblasts foci; AECs II and smooth muscle metaplasia; honeycombing (traction bronchiectasis); moderate inflammatory infiltrate [70,71,72,88,90,92,116].	Lesions depend on model; often homogeneous, inflammation-driven; prevalent NSIP pattern in early stages: interstitial, peribronchiolar fibrosis and inflammation; AECs I degeneration; AECs II hyperplasia; histologic findings more closely resemble UIP in BLM-induced IPF by repeated doses, and aging, genetically modified/humanized mice (honeycombing, subpleural fibrosis, EMT) [74,82,93,94,97,117,118,119].

EndMT: endothelial–mesenchymal transition. EMT: epithelial–mesenchymal transition. NSIP: nonspecific interstitial pneumonia. PH: pulmonary hypertension. UIP: usual interstitial pneumonia.

**Table 2:** Translational strengths, limitations, and applications of selected IPF models.

Model	Major Strengths	Major Limitations	Translational Value and Main Applications
Human	Clinical relevance.	Limited mechanistic experimentation.	Reference, clinical studies, biomarkers.
Dog	Naturally occurring chronic disease, multifactorial etiology, clinical imaging, serial follow-up over time, ethical advantages.	Limited standardized characterization and case numbers.	Comparative pathology, biomarker studies, translational imaging, veterinary clinical research, drug trials.
Cat	Naturally occurring fibrosis, multifactorial etiology, closely resembling UIP pattern, clinical imaging, serial follow-up over time, ethical advantages.	Limited standardized characterization and case numbers.	Comparative pathology, spatial and temporal heterogeneity, biomarker studies, translational imaging, veterinary clinical research, drug trials.
Mouse, rat (chemically induced)	Controlled experiments, reproducibility, mechanistic studies.	Artificial injury, limited chronicity, limited clinical and imaging comparability.	Pathogenesis, molecular pathways, proof-of-concept theranostic target validation and drug screening.
Mouse (genetical engineering/humanized models)	Controlled experiments, aging/genetic susceptibility.	Genetic manipulation, limited availability, partial phenotype.	Mechanistic studies of fibrosis susceptibility, theranostic target validation and drug screening.

UIP: usual interstitial pneumonia.

## Data Availability

No new data were created or analyzed in this study. Data sharing is not applicable.

## Abbreviations

The following abbreviations are used in this manuscript:

IPF	Idiopathic Pulmonary Fibrosis
ILD	interstitial lung disease
PDE4	phosphodiesterase-4
HRCT	high-resolution computed tomography
UTE	ultrashort echo time
MRI	magnetic resonance imaging
PET	positron emission tomography
UIP	usual interstitial pneumonia
AEC II	alveolar epithelial cells type 2
ECM	extracellular matrix
EMT	epithelial–mesenchymal transition
AE-IPF	acute exacerbations of IPF
TGF-β	transforming growth factor-beta
PDGF	platelet-derived growth factor
UK	United Kingdom
USA	United States of America
AI	artificial intelligence
OCT	optical coherence tomography
TLCB	transbronchial lung cryobiopsy
SLB	surgical lung biopsy
PH	pulmonary hypertension
EndMT	endothelial–mesenchymal transition
WHWTs	West Highland White Terriers
IL	Interleukin
NF-κB	nuclear factor kappa B
TNF	tumor necrosis factor
BLM	Bleomycin
CPSF7	polyadenylation factor 7
SDHAF2	succinate dehydrogenase complex assembly factor 2
FAP	fibroblast activation protein
SARS-CoV-2	Severe Acute Respiratory Syndrome Coronavirus 2
NSIP	nonspecific interstitial pneumonia
LTBP	latent TGF-β binding protein
scRNA-seq	single-cell RNA sequencing
BALF	bronchoalveolar lavage fluid
SPP1	Osteopontin
NOD	nonobese diabetic
SCID	severe combined immunodeficiency
KO	Knockout
RAGE	Receptor for Advanced Glycation End-products
RLX	Relaxin
HU	Hounsfield units
MALDI-MSI	Matrix-assisted laser desorption/ionization mass spectrometry imaging
FITC	fluorescein isothiocyanate
IT	Intratracheal
p-Smad	phospho-Smad
OA	oropharyngeal aspiration
μCT	microcomputed tomography
HYP	Hydroxyproline
IP	Intraperitoneal
IV	Intravenous
PFT	pulmonary function test
PAI-1	Plasminogen Activator Inhibitor-1
TEM	Transmission electron microscopy
AEC I	alveolar epithelial cells type 1
SpO_2_	oxygen saturation
HIF	hypoxia-inducible factor
NR4A1	Nuclear receptor subfamily 4 group A member 1
E2	17-β-estradiol
ERα	estrogen receptors α
FAPI	fibroblast-activating protein inhibitor
SUVR	standardized uptake value ratio
α-SMA	α-smooth muscle actin
iPSCs	induced pluripotent stem cells
CCSP	Clara cell secretory protein
AF-1	Antiflammin-1
CSP7	7-amino acid fragment of Caveolin-1 scaffolding domain peptide
IN	Intranasal
mDASCs	mouse distal airway stem/progenitor cells
PDPN	Podoplanin
HOPX	homeodomain-only protein homeobox
AQP5	aquaporin 5
PEEP	positive end-expiratory pressure
PDE4B	phosphodiesterase 4B
cAMP	cyclic adenosine monophosphate
GPCR	G-protein coupled receptor
CREB	cAMP response element binding
MMP7	Matrix Metalloproteinase-7
SAECs	small airway epithelial cells
FVC	forced vital capacity
DLCO	diffusing capacity for carbon monoxide
rMCs	resident mesenchymal cells
Cthrc1^+^	Collagen Triple Helix Repeat Containing 1-positive
aMYFs	activated myofibroblasts
SCID/bg	severe combined immunodeficiency/beige
NSG	NOD/SCID IL2Rγ^−^/^−^
CXCR4	C-X-C chemokine receptor type 4
GvHD	graft-versus-host disease
NOG	NOD.Cg-*PrkdcscidIL2γgtm1 Sug*/JicKoat
SFTPC	Surfactant Protein C
TMX	Tamoxifen
IFN	Interferon
ILC2s	group 2 innate lymphoid cells
IL1RL1	IL-1 receptor-like 1
